# Steroid hormone ecdysone deficiency stimulates preparation for photoperiodic reproductive diapause

**DOI:** 10.1371/journal.pgen.1009352

**Published:** 2021-02-02

**Authors:** Shuang Guo, Zhong Tian, Qing-Wen Wu, Kirst King-Jones, Wen Liu, Fen Zhu, Xiao-Ping Wang

**Affiliations:** 1 Hubei Key Laboratory of Insect Resources Utilization and Sustainable Pest Management, College of Plant Science and Technology, Huazhong Agricultural University, Wuhan, PR China; 2 Department of Biological Sciences, University of Alberta, Edmonton, Alberta, Canada; University of Kentucky, UNITED STATES

## Abstract

Diapause, a programmed developmental arrest primarily induced by seasonal environmental changes, is very common in the animal kingdom, and found in vertebrates and invertebrates alike. Diapause provides an adaptive advantage to animals, as it increases the odds of surviving adverse conditions. In insects, individuals perceive photoperiodic cues and modify endocrine signaling to direct reproductive diapause traits, such as ovary arrest and increased fat accumulation. However, it remains unclear as to which endocrine factors are involved in this process and how they regulate the onset of reproductive diapause. Here, we found that the long day-mediated drop in the concentration of the steroid hormone ecdysone is essential for the preparation of photoperiodic reproductive diapause in *Colaphellus bowringi*, an economically important cabbage beetle. The diapause-inducing long-day condition reduced the expression of ecdysone biosynthetic genes, explaining the drop in the titer of 20-hydroxyecdysone (20E, the active form of ecdysone) in female adults. Application of exogenous 20E induced vitellogenesis and ovarian development but reduced fat accumulation in the diapause-destined females. Knocking down the *ecdysone receptor* (*EcR*) in females destined for reproduction blocked reproductive development and induced diapause traits. RNA-seq and hormone measurements indicated that 20E stimulates the production of juvenile hormone (JH), a key endocrine factor in reproductive diapause. To verify this, we depleted three ecdysone biosynthetic enzymes via RNAi, which confirmed that 20E is critical for JH biosynthesis and reproductive diapause. Importantly, impairing *Met* function, a component of the JH intracellular receptor, partially blocked the 20E-regulated reproductive diapause preparation, indicating that 20E regulates reproductive diapause in both JH-dependent and -independent manners. Finally, we found that 20E deficiency decreased ecdysis-triggering hormone signaling and reduced JH production, thereby inducing diapause. Together, these results suggest that 20E signaling is a pivotal regulator that coordinates reproductive plasticity in response to environmental inputs.

## Author summary

Developmental arrest pervades organismal development and physiology where it facilitates an enormous range of adaptive responses to stressful conditions. Many animals exhibit various forms of developmental arrest that ensures survival under the most adverse environments. Reproductive diapause occurs when adults temporarily suspend reproduction in response to environmental stress and has been documented for a variety of invertebrates, particularly insects. Endocrine signals play a central role in translating environmental cues such as photoperiod into reproductive diapause-related physiology and behavior. However, it has been an unresolved issue as to which endocrine factors can respond to photoperiodic inputs and regulate diapause outputs. In this study, we found that a decrease in ecdysone levels is critical for reproductive diapause to occur. Also, ecdysone could interact with juvenile hormone to regulate the occurrence of reproductive diapause in response to photoperiodic cues. Our findings provide new insight into endocrine mechanisms of photoperiodic reproductive diapause and an example of phenotypic plasticity in animals.

## Introduction

Under suboptimal conditions, many organisms, including nematodes, crustaceans, insects, fish and even mammals, can extend their lifespan and survive through diapause, a plastic and adaptive process that is a form of dormancy [1, 2]. Diapause is a programmed development arrest stage and induced by seasonal environmental cues. Individuals undergoing diapause generally exhibit substantial fat accumulation, reduced metabolism, slowed aging, and enhanced tolerance to adverse conditions [1]. Hence, animals capable of entering diapause can be employed in dissecting mechanisms of obesity, metabolic regulation, and longevity [3–6]. Insects are the most numerous and diverse group of animals. Considering their significant importance on agricultural production and human health, and the advantage of convenient genetic manipulations, insects can serve as the excellent models in the study of diapause [7].

In insects, reproductive diapause is characterized as an arrest in ovarian development and the storage of huge fat reserves. During the diapause preparation phase (DPP), insects must make the decision to enter diapause and initiate different physiological and behavioral programs prior to the entry into diapause [8]. Endocrine signaling is thought to play a central role in responding to the diapause-inducing environmental cues and direct diapause physiological characters during DPP. It is commonly thought that the absence of JH induces the major aspects of reproductive diapause of females and males [9]. As such, JH (via binding to its intracellular receptor Met) is believed to be the sole hormonal factor mediating reproductive diapause occurrence [10–12]. However, some evidence suggests that JH is not the only endocrine factor in the regulation of reproductive diapause. For example in *Locusta migratoria*, significantly lower ecdysteroid titers were recorded for diapausing females than for nondiapausing females [13], a result that is consistent with studies in males of *Pyrrhocoris apterus*. In these bugs, diapausing males have a lower level of makisterone A (a kind of ecdysteroids) than non-diapausing males [14]. These observations raised the idea that the steroid hormone 20-hydroxyecdysone (20E), one of the major active ecdysteroids *in vivo*, may also modulate diapause fate of the adult in response to external environmental cues. Since these studies hinted at a potential role for 20E in reproductive diapause control, we set out to test this idea more rigorously.

20E binds to the intracellular ecdysone receptor (EcR) and then induces the formation of receptor heterodimer to trigger signal cascade for the regulation of molting, metamorphosis, and reproduction [15]. Ecdysone (the precursor of 20E) is produced in the prothoracic gland during larval stages, while it is predominantly biosynthesized in the ovaries of females during the adult stage [16]. In the 1970s, the discovery that adults could synthesize 20E from ovaries raised the possibility that 20E signaling could be involved in the regulation of reproduction [17]. A positive correlation between reproduction and 20E signaling has been established unambiguously in the Dipteran, Lepidopteran and several Coleopterans, such as *Aedes aegypti* [18], *Bombyx mori* [19] and *Tribolium castaneum* [20]. Intriguingly, a pivotal role of 20E has been highlighted in the various reproductive processes of *Drosophila melanogaster* with diapause traits [21]. However, whether 20E signaling could regulate reproductive diapause remains unclear.

20E signaling interacts with JH signaling during several developmental processes, which encouraged us to investigate the potential relationship between 20E and JH in reproductive diapause [22]. Studies on *B*. *mori* revealed that 20E acquires a critical role in the regulation of the transcription of JH biosynthetic genes during the juvenile stage [23]. In mosquito pupae, 20E acts as a developmental signal to directly activate the corpora allata, a JH-producing tissue [24]. More recently, it was found that 20E regulates the ecdysis-triggering hormone (ETH) via 20E receptor (ecdysone receptor, EcR) and thus regulating the JH level indirectly [25]. These studies establish the relationship between 20E signaling and JH production during metamorphosis and reproduction, but whether these hormones interact in photoperiodic reproductive diapause remains poorly understood.

In this study, we have chosen to tackle the roles of 20E during DPP using the cabbage beetle *C*. *bowringi*, a serious pest of cruciferous vegetables in Asia. Under the long-day (LD) condition (16L:8D), beetle females entered reproductive diapause and showed a state of reproductive arrest and accumulated higher amounts of lipids in the fat bodies, whereas the short-day (SD) condition (12L:12D) promoted the development of ovaries and induced reproduction [10, 26, 27]. It has been demonstrated that the absence of JH signaling induced diapause in this beetle and dramatically regulated diapause preparation. Hence, these obvious physiological characteristics of two developmental phases signify that this beetle is an ideal research model for studying the effects of endocrine signaling on reproductive diapause-associated changes during DPP. Here, we found that 20E signaling was suppressed by the LD condition. The inhibited 20E signaling blocked ovarian development but induced the accumulation of huge fat reserves during DPP before diapause initiation. 20E could activate ETH signaling for diapause preparation via the EcR-mediated canonical pathway. Meanwhile, 20E also regulated diapause preparation in a JH-independent manner. This work reveals a remarkable role of 20E in reproductive diapause preparation and establishes a model of the interaction between 20E and JH during DPP.

## Results

### LD-treatment suppressed 20E signaling in female *C*. *bowringi* adults

**Fig 1:**
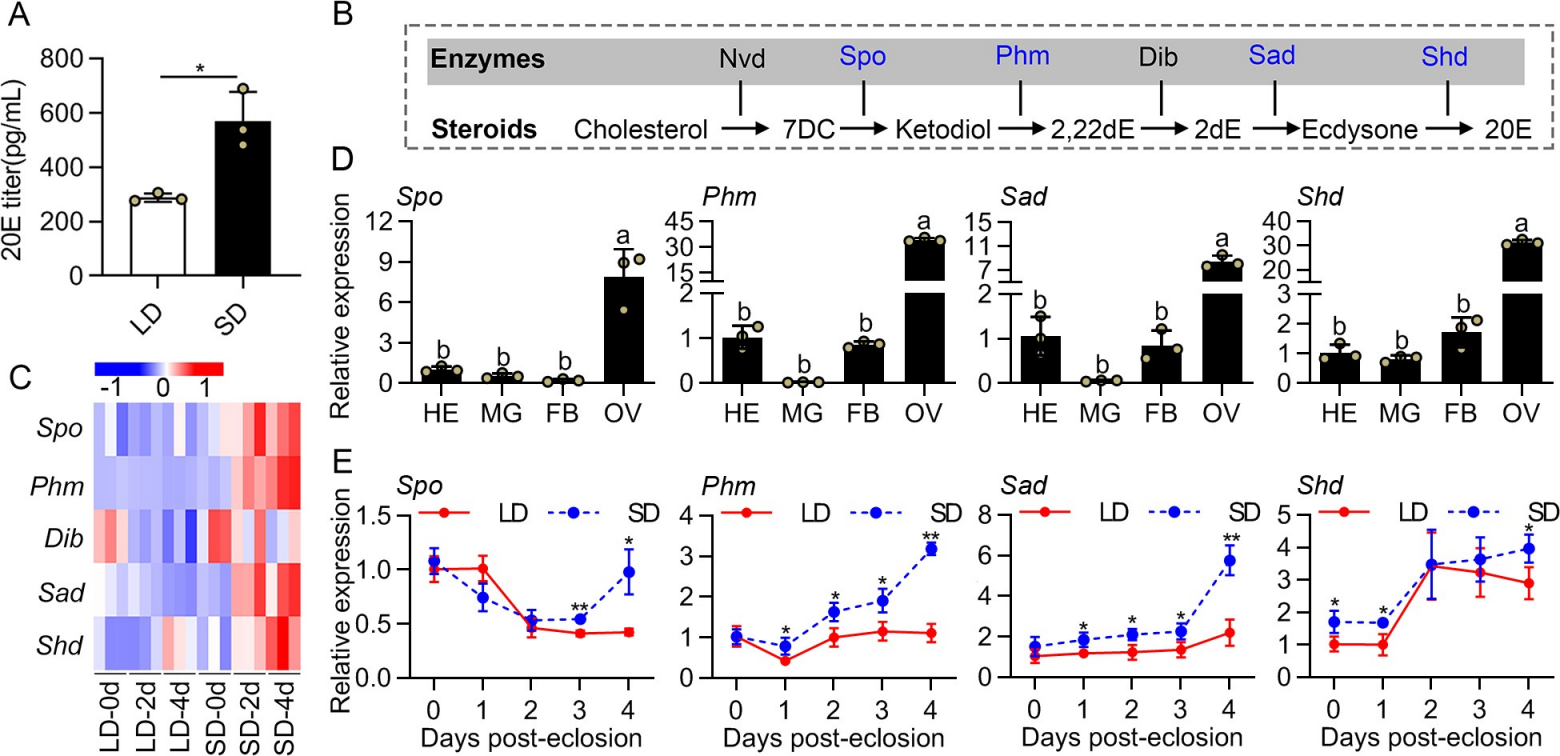
Changes in 20E production in the female adults of *C*. *bowringi* under photoperiodic conditions. (A) Determination of 20E titer in the hemolymph of the LD- and SD-induced *C*. *bowringi* at 4 days post eclosion (PE). (B) 20E biosynthesis pathway in insects. (C) The heat map of expression patterns of 20E biosynthetic genes at 0, 2 and 4 days PE in the LD- and SD-induced females. (D) Tissue expression patterns of *Spo*, *Phm*, *Sad* and *Shd* in SD-induced females at 4 days PE. HE, head; MG, midgut; FB, fat body; OV, ovary. The relative gene expression levels in tissues are presented as fold changes compared to the heads. (E) Determination of mRNA levels of *Spo*, *Phm*, *Sad* and *Shd* in the ovaries of the LD- and SD-induced females from 0 to 4 days PE. The relative expressions of genes at different time points are presented as fold changes compared to the LD females at 0 days PE. Different letters above bars indicate significant between-group differences determined by one-way ANOVA followed by Tukey’s LSD test (α = 0.05). Error bars represent the sd. Asterisks indicate significant differences determined by an Independent-Samples t-test. *P < 0.05, **P < 0.01. Nvd, neverland; Dib, disembodied.

In order to ascertain the basic role of 20E in reproductive diapause of female *C*. *bowringi*, we measured 20E concentrations in the hemolymph of females under LD (diapause) and SD (reproduction) conditions, respectively, at 4 days post-eclosion (PE). The result showed that 20E titers were significantly lower in LD-treated females compared to SD-treated females (Fig 1A). To assess the expression profiles of genes associated with 20E biosynthesis (“Halloween genes”), we analyzed *Spook* (*Spo*), *Phantom* (*Phm*), *Disembodied* (*Dib*), *Shadow* (*Sad*) and *Shade* (*Shd*) (Fig 1B) [28]. First, we generated a heat map for these five Halloween genes, which based on previously published whole body-transcriptome data corresponding to 0, 2 and 4 days PE in LD- and SD-treated females [29] (S1 Table). The mRNA levels of the Halloween genes exhibited a gradual downregulation in LD-treated females at both 2 and 4 PE days (Fig 1C), which is consistent with a reduced 20E titer in LD females. We further characterized the expression patterns of *Spo*, *Phm*, *Sad* and *Shd* by qRT-PCR. These genes clearly showed the ovary-specific expression profiles in the females under SD condition (Fig 1D), consistent with the idea that the ovary is the principal source of 20E during adult reproduction of *C*. *bowringi*. We then examined the expression profiles of these four genes from 0 to 4 days PE in the ovaries of the females under LD and SD conditions using qRT-PCR (Fig 1E), which showed that *Phm*, *Sad* and *Shd* continuously increased in SD females during this time window, and showed overall lower expression in LD females (Fig 1E). The transcript levels of *Spo* in the ovaries gradually rose after 2 days PE, suggesting it may be involved in the female reproductive cycle (Fig 1E). These results strongly suggest that female *C*. *bowringi* have lower 20E levels during DPP.

### Manipulating 20E signaling in the females affected photoperiodic ovarian development

**Fig 2:**
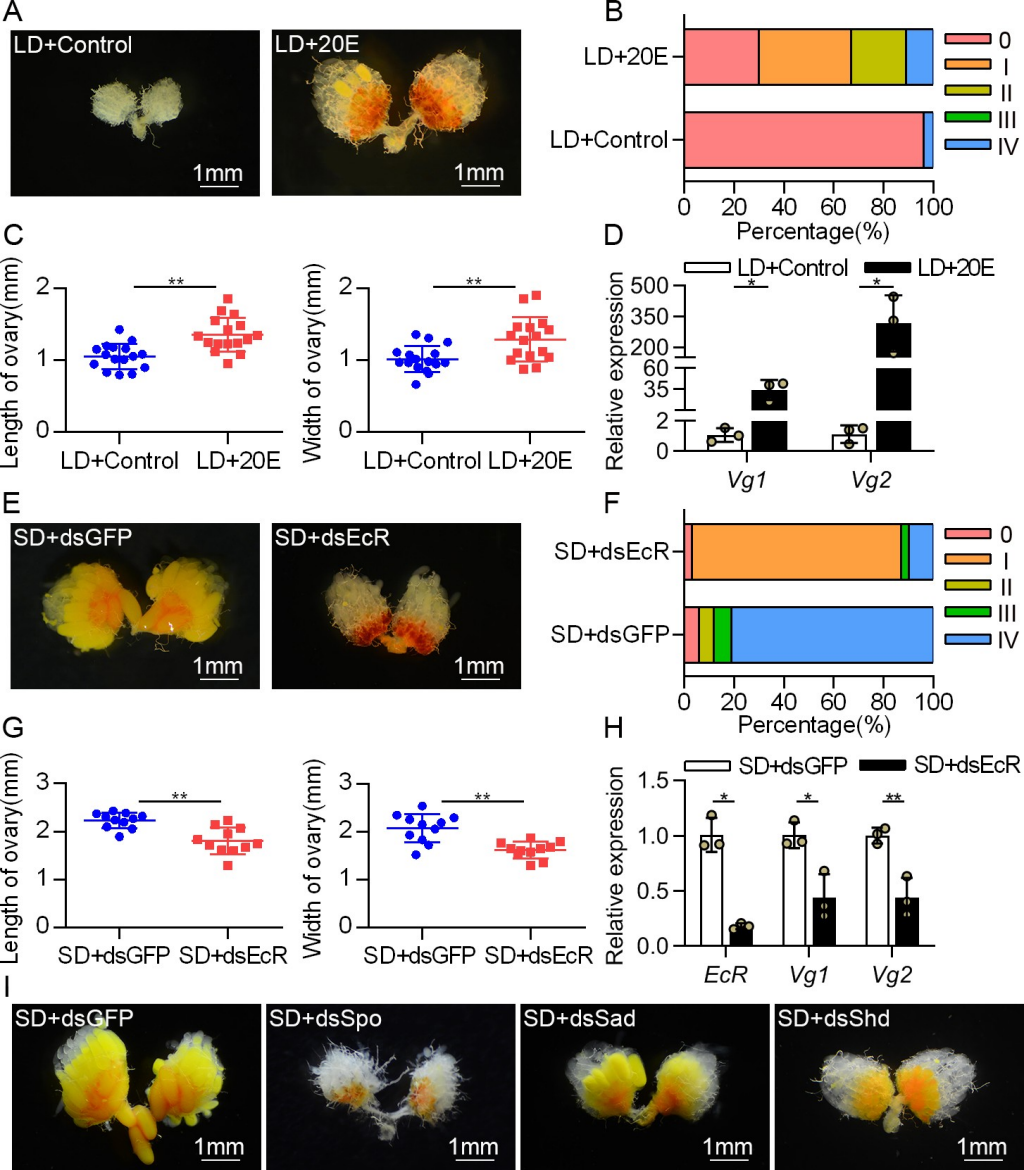
20E signaling promotes ovarian development under SD condition. (A) The LD-induced females were injected with 1μg of 20E at 0 days PE, and the induction of reproduction in response to 20E signaling was tested after 4 days of injection. (B) The development grades and (C) sizes of ovaries were analyzed after treated with 20E. (D) Effect of 20E injection on the expression of *Vg1* and *Vg2* mRNA in the fat bodies. The relative gene expression levels in LD+20E sample are presented as fold changes compared to LD+Control. (E) The reproductive females were microinjected with dsGFP or dsEcR at 0 days PE, and representative phenotypes of ovaries were imaged by a stereomicroscope at 4 days PE. (F) The development grades and (G) sizes of ovaries were analyzed after treated with dsEcR. (H) Relative abundance of *EcR*, *Vg1* and *Vg2* mRNA in the fat bodies after treated with dsEcR. The relative gene expression levels in SD+dsEcR sample are presented as fold changes compared to SD+dsGFP. (I) Representative examples of ovarian development were determined on the 4^th^ day after 20E biosynthetic genes’ RNAi in SD-induced females. Error bars represent the sd. *P < 0.05, **P < 0.01.

To test the regulation of 20E signaling on the preparation of reproductive diapause, we treated the LD-treated females with exogenous 20E at 0 days PE (newly emerged without feeding) and analyzed the phenotypes of the ovaries at 4 days PE. Compared with the solvent control, 20E induced ovarian development and the expression levels of *vitellogenin 1* (*Vg1*) and *Vg2* (Fig 2A–2D). These results suggested deficiency of 20E signaling during DPP leads to reproductive arrest. To confirm this, we decreased 20E signaling by knocking down the 20E receptor *EcR* in SD-induced females. We isolated two cDNAs encoding EcR (EcRA and EcRB) based on the transcriptome data of *C*. *bowringi*, and their common region was used as the RNAi target to perform the following experiments (S1A Fig). Compared with the dsGFP control, the injections of dsRNAs targeting on three independent fragments of *EcR* (dsEcR, dsEcR-2 and dsEcR-3) clearly reduced the expression of *EcR* and showed similar ovarian defects, including the inhibition of yolk deposition and ovarian growth (Figs 2E–2H and S1B and S1C). The expression levels of *Vg1* and *Vg2* in the fat bodies with *EcR* RNAi were reduced by approximately 50% compared with the dsGFP control. Reproductive defects in the SD-induced females with 20E biosynthetic genes’ RNAi were highly similar to that of *EcR* RNAi treatment (Figs 2I and S2A–S2C). There was a significant decrease in ovary size and vitellogenesis. Taken together, these data suggested that 20E-bound EcR promotes ovarian development in SD females and that the reduced 20E signaling triggers reproductive arrest in the LD females during DPP.

### Increasing 20E signaling in LD females restricted lipid accumulation during diapause preparation

**Fig 3:**
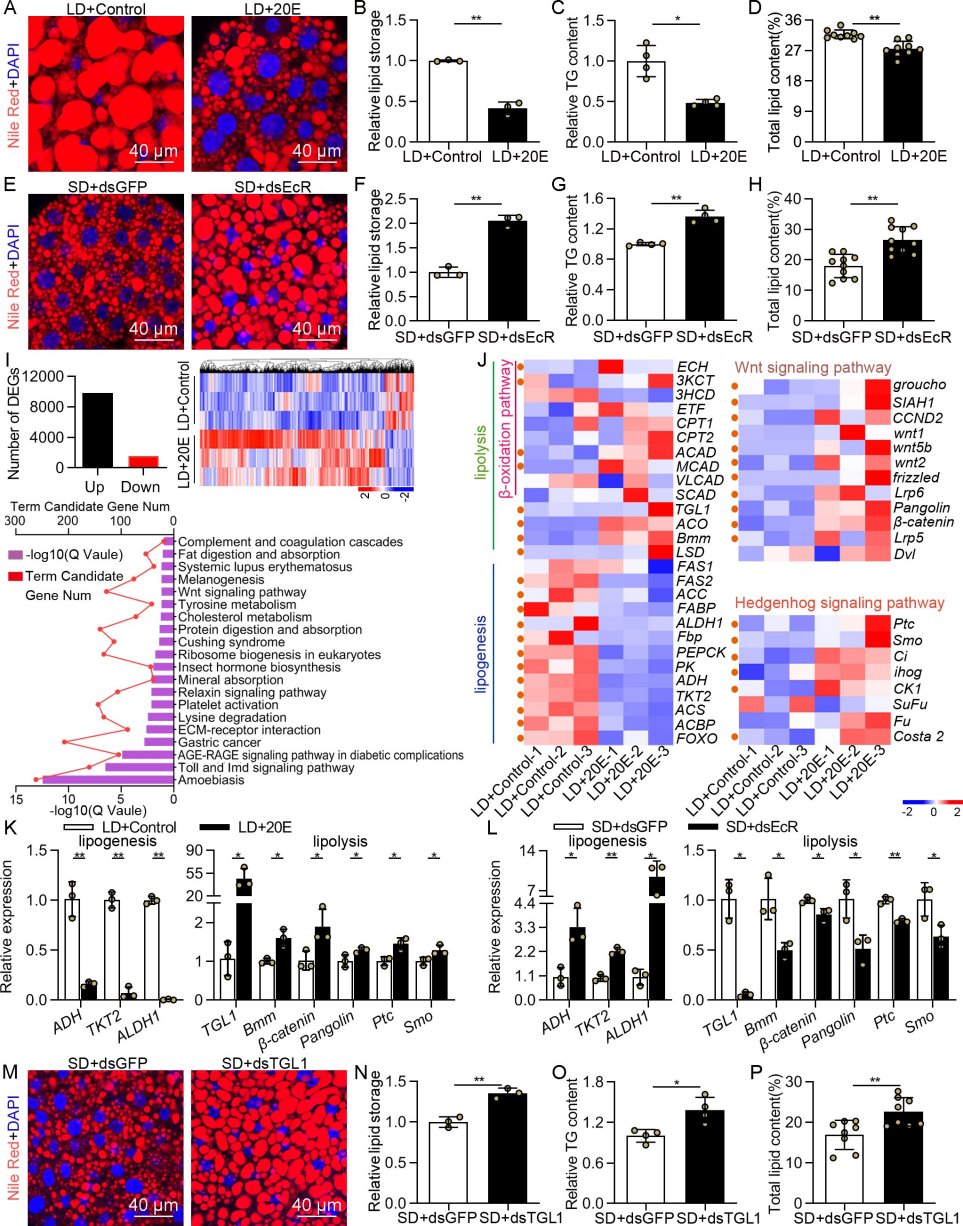
20E signaling suppresses lipid accumulation of diapause preparation. (A) Nile red staining was performed in the fat bodies of the LD-induced female adults after 4 days of 20E injection and (B) relative intensity of fluorescence of lipid droplets quantitatively was analyzed by using ImageJ. Meanwhile, the (C) relative triglyceride (TG) content and (D) total lipid content were detected in the whole body at 4 days PE. At 0 days PE, the reproductive females were microinjected with dsGFP or dsEcR to study the lipid accumulation in the fat body at 4 days PE using (E) Nile red staining and (F) relative intensity of fluorescence of lipid droplets quantitatively analyzed by using ImageJ. Statistical analysis for (G) relative TG content and (H) total lipid content after *EcR* RNAi knockdown. (I) The numbers of DEGs, expression patterns of DEGs and top 20 KEGG pathways were displayed. (J) Heat map representing transcripts of genes involved in lipolysis and lipogenesis at the 2^nd^ day after 20E injection in the LD-induced females. Orange dots represent the DEGs. Transcripts of lipogenesis and lipolysis genes were determined in the fat bodies at the 4^th^ days after (K) 20E injection and (L) *EcR* knockdown. Relative gene expression levels represent the fold changes between (K) LD+Control and LD+20E samples and (L) SD+dsGFP and SD+dsEcR treatments. (M) Nile red staining of fat bodies and (N-P) quantitative analysis of fat contents after *TGL1* RNAi. Error bars represent the sd. *P < 0.05, **P < 0.01.

Besides a cessation of reproduction, sequestration of fat reserves is another conspicuous feature of diapausing insects, since individuals undergoing diapause face increased energy demands [2]. Thus, we examined lipid accumulation in the fat bodies of the LD-induced females after 20E treatment. The results showed that 20E injection profoundly reduced the size of lipid droplets (Fig 3A), and TG and total lipid contents (Fig 3B–3D). Meanwhile, in *EcR*-depleted females, the size of lipid droplets significantly increased (Fig 3E), which is in agreement with current data showing lipid content increased in such females (Fig 3F–3H). We also found that disruption of 20E biosynthesis leads to raised TG content (S2D Fig). Thus, 20E signaling is implicated as a negative regulator of lipid accumulation in photoperiodic reproductive diapause.

To understand further the pathways downstream of 20E-regulated diapause that is repressed in lipid storage, we performed transcriptome analysis using the fat bodies of LD-induced females after 20E treatment. Three biological replicates of RNA-sequencing (RNA-seq) were constructed for each treatment. In total, we obtained 11358 differentially expressed genes (DEGs), of which 9815 were up-regulated and 1543 were down-regulated after 20E application (Fig 3I). DEGs in the transcriptome were mapped to a total of 337 KEGG pathways (S2 Table), the top 20 of which are shown in Fig 3I. DEGs were found to be linked to some lipid metabolic pathways, for instance, cholesterol metabolism, tyrosine metabolism and fat digestion and absorption, suggesting the involvement of 20E in regulation of lipid metabolism during DPP (Fig 3I). We next investigated the expression levels of genes associated with lipolysis such as β-oxidation pathway [30], hedgehog (hh) signaling pathway [31] and wnt signaling pathway [32] and lipid synthesis-related genes including *fatty acid synthase* [33]. There are 25 and 12 DEGs in the lipolysis and lipogenesis process, respectively (Fig 3J). This analysis clearly revealed that 20E application on the LD-induced females dramatically increased transcript levels of genes involved in lipolysis, while genes acting in lipogenesis were significantly downregulated (Fig 3J and S3 Table). qRT-PCR analysis of selected critical genes also confirmed this result (S3 Fig).

Our previous studies have identified a series of genes which showed a potential to regulate lipogenesis and lipolysis during the DPP. *Alcohol dehydrogenase NADP* (+) (*ADH*), *transketolase 2* (*TKT2*) and *retinal dehydrogenase 1* (*ALDH1*) are critical genes involved in lipogenesis, whereas *triacylglycerol lipase 1* (*TGL1*) could induce lipolysis [33–36]. *Brummer* (*Bmm*), hh signaling pathway and wnt signaling pathway have been reported to play crucial roles in regulating lipid and energy metabolism [31, 32, 37]. In addition, transcriptome analysis confirmed that these genes are DEGs. Therefore, we measured the mRNA levels of the above genes and other key genes involved in these two pathways (*Patched* (*Ptc*), *Smoothened* (*Smo*), *Pangolin* and *β-catenin*) after 20E treatment and *EcR* RNAi depletion to evaluate the role of 20E signaling in lipid metabolism control. The expression levels of *ADH*, *TKT2* and *ALDH1* were significantly lower in the 20E injection group relative to controls (Fig 3K); conversely, the expression levels of *TGL1*, *Bmm*, *Ptc*, *Smo*, *Pangolin* and *β-catenin* were higher relative to the controls (Fig 3K), suggesting the critical role of 20E in suppression of lipid accumulation. Meanwhile, the expression levels of *ADH*, *TKT2* and *ALDH1* were remarkably upregulated after *EcR* RNAi, whereas that of *TGL1*, *Bmm*, *Ptc*, *Smo*, *Pangolin* and *β-catenin* were significantly downregulated (Fig 3L). To confirm the candidates involved in the 20E-regulated lipid storage in photoperiodic diapause, we selected *TGL1* as an example and validated its function in the regulation of reproductive diapause. The results showed that *TGL1* was predominantly expressed in the fat bodies of females destined for reproduction but exhibited a very low expression in females destined for diapause (S4A and S4B Fig). When we knocked down *TGL1* in the reproductive females (S4C Fig), individuals showed a diapause-like trait, the large accumulation of fat (Fig 3M–3P). Together, EcR-mediated 20E signaling suppresses lipid storage of diapause preparation by modulating the expression of genes associated with lipogenesis and lipolysis.

### 20E signaling promoted JH production and showed a negative role in reproductive arrest

**Fig 4:**
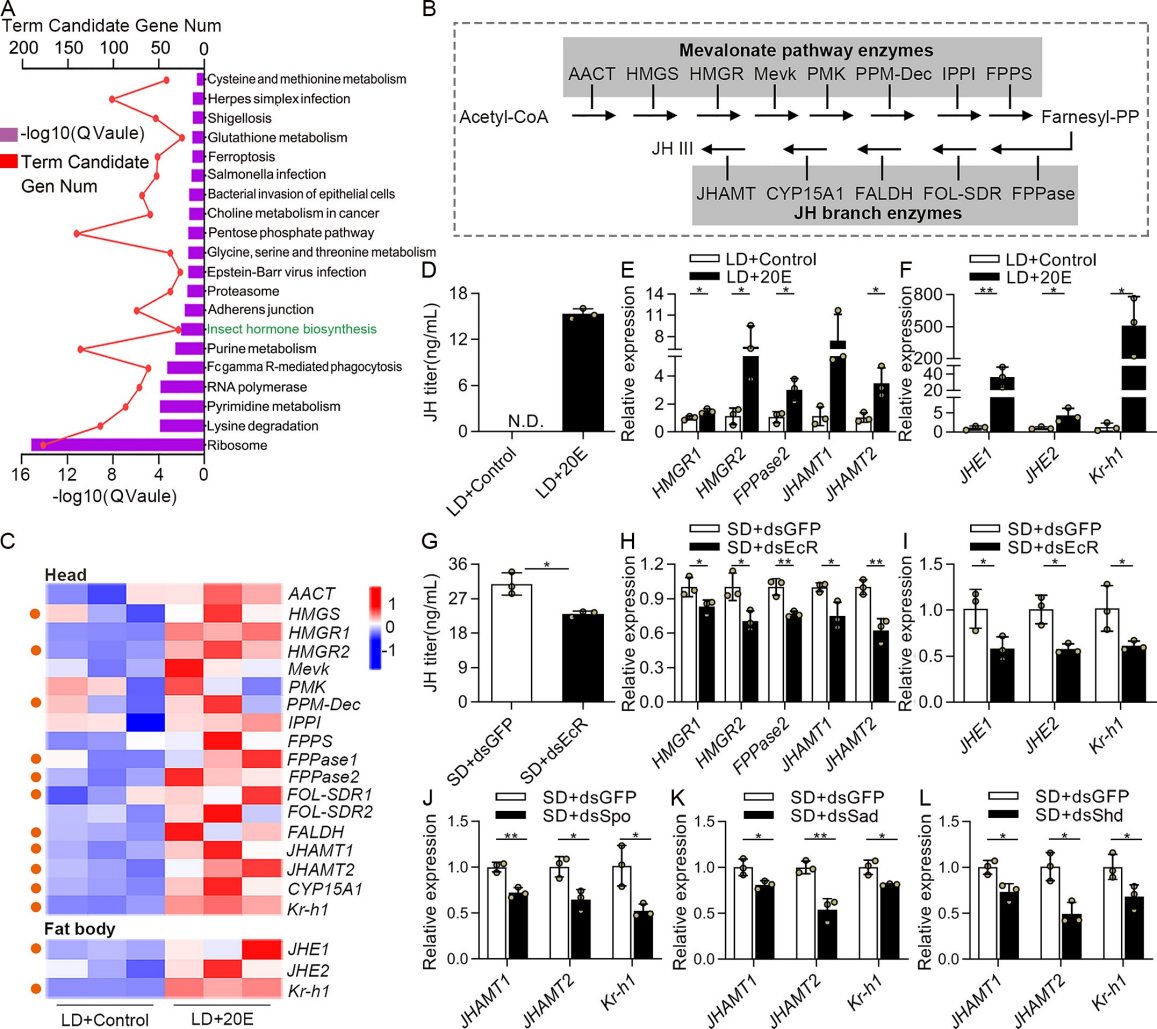
Effects of 20E signaling on the JH signaling in the female adults of *C*. *bowringi* under photoperiodic conditions. (A) Top 20 pathways identified in KEGG pathway analysis in DEGs. (B) JH biosynthesis pathway in insects, which is divided into early (mevalonate pathway) and late (JH branch) steps. (C) The heat map represented transcripts of JH biosynthesis and JH-inducible genes at the 2^nd^ days after 20E injection in the LD-induced females. Orange dots represent the DEGs. (D) JH titer measurement in the hemolymph of the LD-induced female adults at the 4^th^ days after 20E injection, as determined by LC-MS/MS. N.D., not detected. (E) Expression levels of JH biosynthetic genes were detected in the heads at 4 days PE after injection with 20E. (F) The relative abundance of JH-inducible genes mRNA in the fat bodies after treated with 20E injection. Relative gene expression values in the LD+20E sample are presented as fold changes compared to LD+Control. (G) Detection of JH titer in the hemolymph of SD-female 4 days after injection with either dsGFP or dsEcR on the day of eclosion. (H) The expression of JH biosynthetic genes in the heads were analyzed by qRT-PCR after *EcR* RNAi. (I) The differential expression of JH-inducible genes was determined in the fat bodies after dsEcR injection. *JHAMT1*, *JHAMT2* and *Kr-h1* transcript levels in the heads after silencing with (G) *Spo*, (K) *Sad* and (L) *Shd*. Relative gene expression values in the RNAi samples are presented as fold changes compared to the SD+dsGFP control. Error bars represent the sd. *P < 0.05, **P < 0.01.

JH is the primary hormonal factor that regulates reproductive diapause occurrence. Absence of JH induced multiple traits of reproductive diapause in insects, and exogenous JH mimics could restrain reproductive diapause preparation through a conserved Met-dependent pathway in the *C*. *bowringi* [10] and *P*. *apterus* [38]. Therefore, we asked whether 20E could regulate diapause preparation through targeting JH production. To test this, we collected the heads (which contain the JH-producing corpora allata) of LD-treated females after 20E injection for RNA-seq and generated transcript profiles. We noticed that insect hormone biosynthesis pathway was significantly enriched (*P* = 0.0092), determined by KEGG analysis (Fig 4A). Next, we selected 17 genes enriched in JH biosynthesis pathway (Fig 4B) and JH-inducible gene *Krüppel homolog 1* (*Kr-h1*) to generate the heat map for analysis (Fig 4C and S4 Table). Among them, 11 genes were identified as DEGs. Transcripts of these DEGs were observably upregulated in the 20E-treated heads (Fig 4C). We then re-visited the transcriptome data of the fat bodies obtained from 20E-applied females and examined whether injection with 20E affected JH signaling. The transcription levels of *JH esterase 1* (*JHE1*), *JHE2* and *Kr-h1* were detected, as these genes are transcriptionally induced by JH during multiple developmental processes [39, 40]. The results showed transcripts of *JHE1* and *Kr-h1* exhibited a significant elevation after 20E treatment (Fig 4C). The qRT-PCR analysis of selected JH biosynthesis and JH-inducible genes also verified these transcriptome data (S5 Fig). These results suggested that 20E injection in the LD females induced the expression of genes associated with JH biosynthesis and JH signaling.

To verify whether 20E really regulates JH production during photoperiodic reproductive diapause, we used liquid chromatography-tandem mass spectrometry to determine the JH titer at 4 days PE in LD females after 20E treatment. JH titer in the hemolymph collected from LD females without 20E injection (control) was not detectable (N.D.), but exhibited significant levels in LD females that were injected with 20E (Fig 4D), demonstrating 20E indeed promoted JH production. It is well-documented that *HMG CoA reductase 1* (*HMGR1*), *HMGR2*, *farnesyl pyrophosphatase 2* (*FPPase2*), *juvenile hormone acid methyltransferase 1* (*JHAMT1*) and *JHAMT2* are the critical enzymes that synthesize JH [41, 42]. We therefore analyzed the transcript levels of these genes after 20E treatment. Consistent with the JH level data, the changes in expression of *HMGR1*, *HMGR2*, *FPPase2* and *JHAMT2* were obviously enhanced in the 20E injection group when compared with their expression in the control group (Fig 4E). In addition, we also observed 20E application resulted in sharply increased levels of JH-inducible genes expression (Fig 4F). Next, we tested whether JH titer and JH signaling were controlled by EcR-mediated 20E signaling. We found that the JH titers and transcript levels of JH biosynthetic genes decreased significantly after injection of dsEcR in SD females (Fig 4G and 4H). Similarly, the repression of JH signaling seen in the *EcR*-depleted females was also evident (Fig 4I). Furthermore, RNAi of 20E biosynthetic genes in the SD-induced females exhibited a tendency towards reduced *JHAMT1*, *JHAMT2* and *Kr-h1* expression levels, further validating that 20E promotes JH production (Fig 4J–4L).

**Fig 5:**
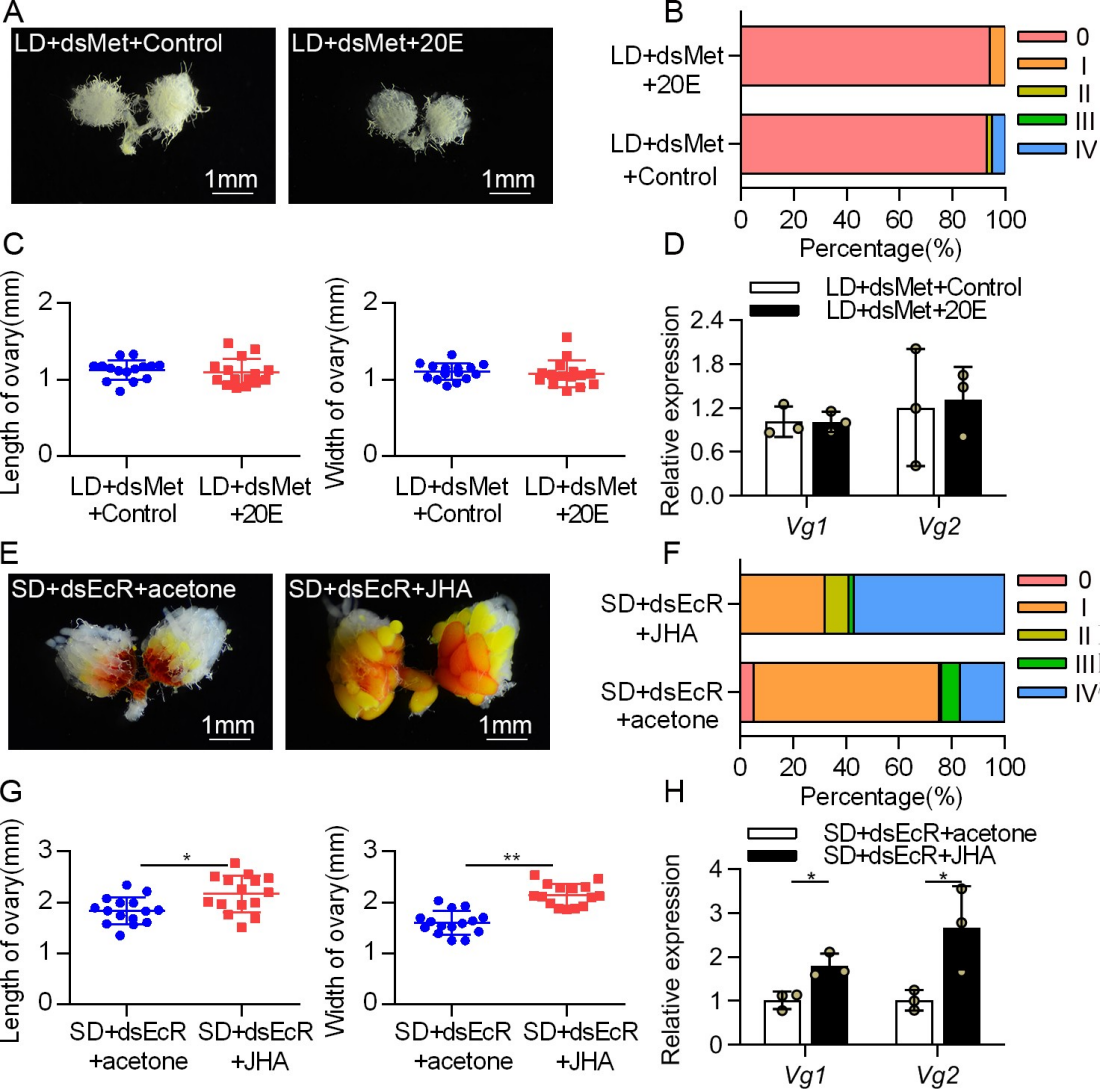
20E promotes SD-induced ovarian development by targeting JH signaling. (A) The LD-induced females were treated with dsMet following a 24 h induction with 1μg 20E to investigate the induction of reproduction in response to 20E-JH signaling. (B) The development grades and (C) sizes of ovaries were analyzed following dual-injection with dsMet and 20E. (D) The expression of *Vg1* and *Vg2* were detected in the fat bodies after injection with dsMet and 20E. Relative gene expression values represent the fold changes between LD+dsMet+Control and LD+dsMet+20E. (E) Representative phenotypes of ovaries from SD females injected with dsEcR and JHA. (F) The development grades and (G) sizes of ovaries were analyzed after treated with dsEcR and JHA. (H) Relative abundance of *Vg1* and *Vg2* mRNA in the fat bodies after injection with dsEcR and JHA. Relative gene expression values represent the fold changes between SD+dsEcR+acetone and SD+dsEcR+JHA. Error bars represent the sd. *P < 0.05, **P < 0.01.

The above data suggest that 20E via *EcR* promotes both JH production and ovarian development, but the direct evidence that 20E-regulated JH signaling modulates reproduction is absent. To address this, we first knocked down the JH receptor *Met* in females that were destined for diapause and applied 20E. We found that 20E-induced yolk deposition, ovarian growth, and *Vg* expression were completely blocked after *Met* depletion (Fig 5A–5D). Next, we tested whether JH could rescue the ovary arrest induced by *EcR* RNAi. Clearly, JHA application removed the block of ovarian development and reproductive gene expression (Fig 5E–5H). The data indicated that in LD females, downregulated 20E signaling reduces JH production and restrains JH-mediated reproductive development, thereby inducing reproduction arrest.

### *ETH* is 20E-responsive, and ETH-RNAi caused reproductive arrest and downregulation of JH signaling

**Fig 6:**
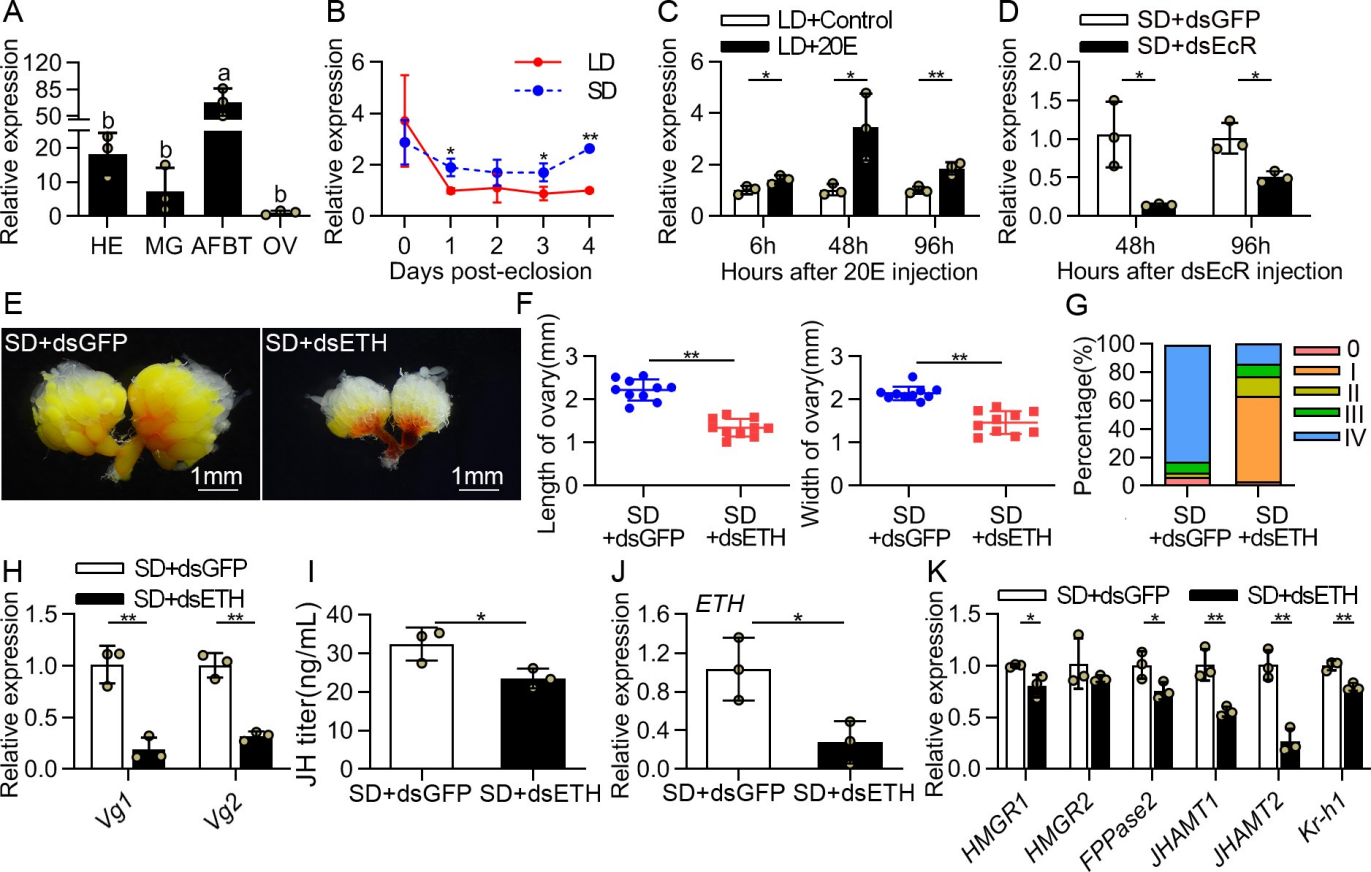
Knocking down *ETH* arrests ovarian development and decreases JH signaling under SD condition. (A) Tissue distribution of *ETH* in SD-induced females at 4 days PE. HE, head; MG, midgut; AFBT, abdominal fat bodies attached with trachea; OV, ovary. Relative gene expression values in the tissue samples are presented as fold changes compared to the ovary samples. (B) Patterns of *ETH* expression detected by qRT-PCR in the in the AFBT tissues on 0–4 days after eclosion. Relative expression levels of genes at different time points are presented as fold changes compared to the LD females at 4 days PE. (C) The transcriptional expression of *ETH* was analyzed after 20E injection for 6, 48 and 96 h in the AFBT tissues of the LD-induced females. (D) qRT-PCR of *ETH* in the AFBT tissues of SD-females injected with dsEcR, analyzed at 48 and 96 h. (E-G) Effect of knockdown of *ETH* in female on ovarian development and ovary length and width. (H) Relative expression levels of *Vg1* and *Vg2* after dsETH injection measured by qRT-PCR in the fat bodies. (I) Reduction of JH titer in SD-females following *ETH* silencing. qRT-PCR measurement of (J) *ETH*, (K) JH biosynthetic genes and *Kr-h1* expression levels were analyzed in the heads after dsETH treatment. The relative gene expression levels in the treatments are presented as fold changes compared to the control groups. Different letters above bars indicate significant between-group differences determined by one-way ANOVA followed by Tukey’s LSD test (α = 0.05). Error bars represent the sd. *P < 0.05, **P < 0.01.

20E, ETH and JH constitute a hormonal network essential for reproductive success in *D*. *melanogaster* [25]. This prompted us to ask whether ETH could interact with 20E and JH, and then contribute to photoperiodic reproductive diapause in *C*. *bowringi*. To gain insight into a possible role of ETH in reproductive diapause preparation, we examined its spatiotemporal distribution in females. Although previous work found that ETH was expressed in Inka cells of the entire tracheal system [43], it was not feasible for us to dissect the tracheal system of *C*. *bowringi* and test *ETH* expression by qRT-PCR. Therefore, we collected heads, midguts, ovaries and abdominal fat bodies attached with trachea (AFBT) and determined the primary pattern of *C*. *bowringi ETH*. This revealed that *ETH* mRNA abundance was higher in the AFBT tissues (Fig 6A). We then compared the profiles of *ETH* in LD and SD females, which showed that *ETH* expression remained stable and lower in the LD-treated females (Fig 6B). This result suggested that lower *ETH* may be essential to reproductive diapause occurrence of *C*. *bowringi*. ETH is known to be under control of 20E during different developmental stages [25]. We therefore measured whether *ETH* expression level was influenced in 20E-treated diapausing females and dsEcR-treated nondiapausing females. At 6 h, 48 h and 96 h post-injection, *ETH* transcript levels were significantly upregulated by 20E (Fig 6C). Correspondingly, *ETH* mRNA levels were decreased by 85% and 50% at 48 h and 96 h post-dsEcR injection, respectively (Fig 6D). These results strongly suggested that *ETH* expression is positively regulated by 20E signaling in *C*. *bowringi* females. We further knocked down *ETH* in SD-induced females and observed clear phenotypes of arrested ovarian development (Fig 6E–6G). *ETH* knockdown reduced *Vg1* and *Vg2* mRNA levels to 18% and 32% of their respective control levels (Fig 6H). The JH titer as well as the expression levels of JH biosynthetic genes and *Kr-h1* were markedly reduced in the *ETH*-depleted females (Fig 6I–6K). Moreover, we knocked down the *ETH receptor* (*ETHR*) to confirm the function of ETH signaling in JH production. Two cDNAs encoding ETHRA (GenBank Accession No. MW291549) and ETHRB (GenBank Accession No. MW291550) were isolated based on the transcriptome data of *C*. *bowringi*, and their common region was used for qRT-PCR amplification and as an RNAi target site (S6B Fig). *ETHR* transcript was highly abundant in the heads (S6A Fig). RNA knockdown of *ETHR* expression resulted in blocked ovary development and decreased JH signaling (S6C–S6H Fig). Overall, these data suggested ETH signaling is essential to link 20E signaling to JH signaling and regulate reproductive diapause preparation of *C*. *bowringi*.

We next investigated whether ETH is sufficient for ovary development by injecting the mature ETH into LD-females. To this end, we first knocked down *ETH* in SD-induced females and injected ETH peptide to test the activity of the synthetic ETH peptide. As expected, the reproductive defects following reduction of *ETH* expression were rescued with ETH peptide injection into SD-females (S7A–S7D Fig). Also, JH signaling was increased after dual injection with dsETH and ETH peptide, compared to the dsETH group alone (S7E Fig). These results not only suggested that synthetic mature ETH peptide is active *in vivo* but also validated the function of ETH in JH-mediated ovary development. However, when we injected the ETH peptide into LD-females, the ovaries in the treatment groups showed no yolk accumulation and were small in size, comparable to what we observed of the control group (S8A Fig). There was no significant difference in ovary sizes between the PBS control and the ETH peptide treatments (S8B Fig). Combined with the RNAi data of *ETH* and *ETHR* in SD-females, this suggested that ETH signaling is essential but not sufficient to trigger JH-mediated ovary development.

### 20E signaling regulated lipid accumulation for diapause preparation in both JH-dependent and -independent manners

**Fig 7:**
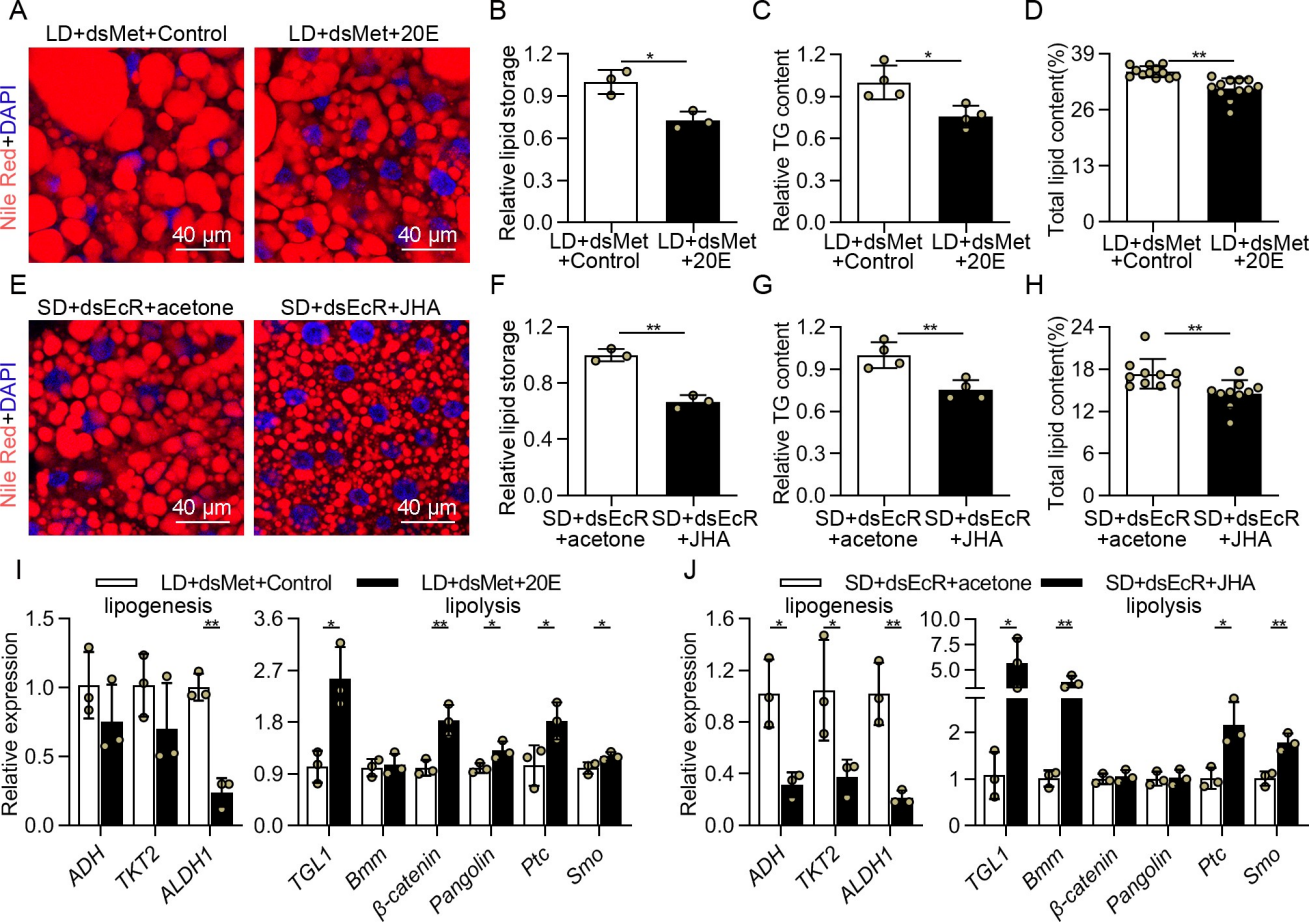
The regulation of lipid accumulation by 20E and JH signaling pathways under photoperiodic conditions. (A) Nile red staining was performed in the fat bodies of the LD-induced female adults after dual-injection with dsMet and 20E. (B) Relative intensity of fluorescence of lipid droplets quantitatively analyzed by using ImageJ. Meanwhile, (C) the relative TG content and (D) total lipid content were detected in the whole body at 4 days PE. (E) At 0 days PE, the reproductive females were microinjected with dsEcR following a 24 h induction with 30 μg of JHA to study the lipid accumulation in the fat body at 4 days PE using Nile red staining. (F) Relative intensity of fluorescence of lipid droplets quantitatively analyzed by using ImageJ. Statistical analysis for (G) relative TG content and (H) total lipid content after dual-injection with dsEcR and JHA. Relative levels of key genes related to lipolysis and lipogenesis transcripts in the fat bodies after (I) dual-injection with dsMet and 20E, as well as (J) dsEcR and JHA. Relative gene expression values in the treatment group are presented as fold changes compared to the control groups. Error bars represent the sd. *P < 0.05, **P < 0.01.

The results above demonstrated that 20E promotes ovarian development through JH signaling, but whether JH signaling is also involved in 20E-regulated lipid accumulation is unclear. Thus, we further analyzed lipid accumulation in the fat bodies of LD-induced females after dual-injection with dsMet and 20E. Application of 20E on *Met*-depleted females evidently reduced the size of lipid droplets in the fat bodies, and TG content and total lipid content in the whole bodies (Fig 7A–7D), suggesting 20E could regulate lipid accumulation without JH signaling. Interestingly, when we consider the results from Fig 3A, the lipid reduction and transcriptional downregulation of lipogenesis genes (Fig 7I), induced by dual injection of dsMet and 20E, is partial and not complete, suggesting that 20E still needs JH to suppress lipid storage. Furthermore, we examined lipid accumulation in the reproductive females of dsEcR further treated with JHA. Compared to control females, application of JHA on *EcR*-depleted females resulted in an apparent suppression of lipid droplet accumulation, resembling the influence of dual-injection with dsMet and 20E in LD-induced females (Fig 7E and 7F). The TG content and total lipid content were significantly decreased relative to control (Fig 7G and 7H). Altogether, we concluded that 20E could repress the lipid accumulation in both JH-dependent and -independent manners.

Next, we analyzed whether 20E affects the expression of the pivotal genes related to lipolysis and lipogenesis identified during the previous step in JH-dependent or -independent manner. The transcript levels of *ALDH1* were significantly decreased and that of *TGL1*, *Ptc* and *Smo*, were significantly increased after 20E injection, although *Met* RNAi could result in JH signaling interruption (Fig 7I). Consistent with this observation, transcripts of *ALDH1* dramatically declined, and then *TGL1*, *Ptc* and *Smo* exhibited an obvious elevation in animals treated with dsEcR and JHA (Fig 7J). This suggested that 20E could regulate these gene expressions in both JH-dependent and -independent manners. Meanwhile, expression levels of *ADH*, *TKT2* and *Bmm* were evidently unchanged after dual-injection with dsMet and 20E (Fig 7I), but *ADH* and *TKT2* downregulated, and *Bmm* upregulated in the SD-induced females of dsEcR further treated with JHA (Fig 7J). Thus, 20E may induce *ADH*, *TKT2* and *Bmm* expressions through JH. Interestingly, *Pangolin* and *β-catenin* were upregulated in the LD-induced females after dsMet and 20E treatment (Fig 7I). However, the levels of *Pangolin* and *β-catenin* had no significant effect after *EcR* RNAi, even in the presence of JHA (Fig 7J). It supported that 20E could target the wnt signaling pathway in the fat body to regulate lipolysis process in a JH-independent manner.

## Discussion

### 20E as a novel endocrine factor regulating reproductive diapause occurrence

Many animals have evolved mechanisms for arresting their development to survive adverse environmental conditions. Diapause is the one common form of developmental arrest. In insects, reproductive diapause is considered as a hormonally regulated developmental arrest state [9]. Understanding how the occurrence of reproductive diapause is precisely regulated by hormonal signals is a long-standing objective in biology. Our results have shown that 20E production is downregulated by reproductive diapause-inducing photoperiod in female adults of *C*. *bowringi*. The reduced expression of genes along the 20E biosynthesis pathway is coordinated with lower 20E titer during DPP, suggesting that 20E deficiency is central to maintaining the photoperiodic reproductive diapause state. The photoperiodic regulation of 20E production is a prerequisite of the involvement of 20E signaling in reproductive diapause, as diapause is induced by environmental cues, especially photoperiods. Although we still know little about the mechanisms by which photoperiods regulate 20E production for reproductive diapause, some work concerning the circadian clock-mediated 20E biosynthesis may provide us with clues. For example, the circadian clock, of which *Timeless* (*tim*) and *Period* (*per*) are key players, can respond to photoperiods and was identified as an important driver for ecdysone production during metamorphosis of *D*. *melanogaster* [44]. This mechanism has not been confirmed in reproductive diapause, but some circadian clock genes, such as *tim* and *per*, have been found to be essential to photoperiodic diapause induction [45]. Several lines of evidence suggest that 20E may function as a prominent coordinator of diapause in larvae and pupae that responds to photoperiodic factors [9]. In *Chymomyza costata* larvae, the long day signal could stimulate the synthesis and release of ecdysteroids while the short day signal may inhibit it. The inhibition of the 20E biosynthetic pathway may represent important early steps in larval diapause induction [46]. Thus, 20E could be a key environmentally responsive developmental signal.

It is well-documented that 20E signaling is involved in regulating multiple reproductive processes [21, 47], but its function in reproductive diapause, the opposite of reproduction, has not been established. Interestingly, several studies suggest that 20E probably regulates the termination of reproduction arrest in some insect species. In the absence of 20E, certain insects may exhibit a cessation of ovarian development, such as *L*. *migratoria* [13] and *P*. *apterus* [14]. Further evidence supports that a role for 20E in reproductive diapause is furnished by the responsiveness of diapausing adults to 20E. Topical application of 20E successfully terminated reproductive diapause of *D*. *melanogaster* and accelerated the yolk protein uptake by oocytes [48]. Also, exogenous 20E induced ovarian development and accelerated vitellogenesis in diapausing *Dermacentor niveus* [49]. These studies focused on the roles of 20E in terminating diapause. However, our work investigated the function of 20E in diapause preparation, which precedes diapause. We found that 20E levels are downregulated before the female *C*. *bowringi* enters diapause. Manipulation of 20E signaling by *EcR* RNAi and exogenous 20E application remarkably affected ovarian development and diapause destiny. Thus, 20E could determine whether insects enter reproductive diapause based on the environmental cues they perceived.

The huge accumulation of energy reserves is another diapause characteristic. Previous studies suggest the potential inhibitory effect of 20E on reproductive diapause, especially concerning the arrest of reproductive development, but there is a lack of evidence that 20E could suppress reproductive diapause by regulating lipid storage. Here, we demonstrated the negative roles of 20E signaling in lipid storage during reproductive diapause preparation. Interestingly, 20E signaling is also considered the pivotal role to regulate the lipid metabolism in insects during various physiological and developmental processes. For example, 20E reduced food consumption leading to fat body lipolysis through activating lipase gene *Bmm* expression during molting and pupation in *B*. *mori* [37]. This finding is according with the previous report, in which *EcR* mutant resulted in lipid accumulation in the fat body, suggesting EcR-mediated 20E signaling is essential in the lipid metabolism [50]. It is also found that 20E through EcR promotes lipid metabolism for reproductive development during the post-blood meal phase of *A*. *aegypti* [30]. Hence, 20E signaling may play a common role in fat storage and metabolism during diapause, metamorphosis and reproduction. In addition, transcriptomic analysis showed that transcript levels of genes related to β-oxidation, wnt signaling and hh signaling exhibited a simultaneous elevation after 20E injection, as well as other lipid degradation genes, implying that activation of these pathways under the control of 20E could reduce fat mass during DPP. Support for this idea comes from studies that implicate wnt signaling and hh signaling in adipose tissue biology of *Drosophila* and mammals [31, 32]. Most importantly, 20E acts as a key upstream factor that initiates wnt signaling to regulate photoperiodic pupal diapause in *Helicoverpa armigera* [51], further supporting our hypothesis. Therefore, 20E regulates fat storage for diapause preparation through conserved pathways, and this is accomplished via canonical 20E-EcR signaling.

### 20E regulates photoperiodic reproduction arrest by targeting on JH production

In *A*. *aegypti*, JH plays a priming role in the regulation of *Vg* expression during the pre-blood meal period, while 20E is the central stimulus of post-blood-meal events that induce vitellogenesis in the fat body [52]. Analogously, JH appears to control *Vg* synthesis in the fat body and 20E signaling is required ovarian maturation processes in *T*. *castaneum* [20, 53]. These studies clearly indicate that insect reproductive events are controlled by different types of hormones. In addition, there have been long-standing questions about the role of 20E in the regulation of JH biosynthesis during the reproductive cycle in insects. Many factors stimulate JH production including allatostatins, allatotropins, and neural and hormonal inputs [42]. 20E is considered to be one of these regulators because an increase in 20E titer was sufficient to trigger corpora allata maturation [24]. During the adult stage of *D*. *melanogaster*, 20E could stimulate ETH production and thus regulate JH biosynthesis to maintain normal reproduction [25]. These data imply 20E may interact with JH to regulate insect reproduction. However, how 20E and JH jointly regulates photoperiodic reproductive diapause remains poorly understood. In the present study, we found that injection of 20E in the LD-induced females resulted in a stable, obvious increase of JH titer and JH signaling, in turn, JH titer and JH signaling also exhibited significant reduction after knockdown of *EcR* in the SD-induced females. This coordinated expression related to JH biosynthesis may be an innovative mechanism involved in the crosstalk between 20E and JH in the reproductive plasticity. It is worth mentioning that transcriptomic analysis coupled with 20E application and *EcR* RNAi silencing showed that a highly significant expression changes in most genes of JH biosynthesis after the treatments. Thus, 20E could contribute to turn on the activating program of corpora allata (CA). Studies on mosquitoes and *Manduca sexta* also suggested that 20E has a stimulatory effect on JH synthesis in the CA [24, 54]. The incubation of brain-CA-corpora cardiaca complexes with 20E *in vitro* was sufficient to induce a significant elevation in JHAMT activity [24]. Additionally, 20E also targets the CA to regulate JH biosynthesis through *EcR* [55]. Recent studies demonstrated high expression levels of 20E receptors in the CA, which suggests stimulatory functions of the 20E signaling pathway in the CA [24, 56]. Our study is consistent with this notion, since we found an enrichment of *EcR* transcripts in *C*. *bowringi* heads (S1D Fig). Nevertheless, we propose here that 20E is not the only upstream factor regulating JH production in photoperiodic reproductive diapause, as the JH titer and expression of JH biosynthetic genes remained at a certain level after *EcR* RNAi.

To dissect the mechanism by which 20E regulates JH production for reproductive diapause, we selected ETH as a candidate. Previous evidence indicated that Inka cells, the sole source of ETHs, are distributed over the branch points of the tracheal system [43]. Two pairs of Inka cells in the ventral thorax and seven pairs in the dorsal abdomen were observed throughout adulthood in *Drosophila* [25]. In our study, we found high *ETH* mRNA levels in the abdominal fat bodies attached with trachea. In many insect species, fat body cells weakly adhere to each other, requiring the ramifications of tracheoles to bind one cell with another [57]. This may result in the fat body containing affluent trachea and Inka cells, and raise the expression of *ETH* in the AFBT tissues. Interestingly, we also found that *ETH* exhibited higher expression in the SD females, showing a potential of photoperiodic regulation at the transcriptional level. This could be the basis of *ETH* regulating reproductive diapause. To validate this, we knocked down *ETH* in females destined for reproduction and found that the yolk deposition and ovarian growth were almost completely suppressed, and that these females showed a diapause-like ovary phenotype. Moreover, JH titers were clearly reduced by depleting *ETH*. Our results agree with previous reports that ETH plays a crucial functional role in maintaining JH activity to promote reproduction in females [25]. However, we observed that injection of mature ETH peptide in LD-treated females did not stimulate ovarian growth and yolk deposition. These data suggested that ETH signaling is essential but not sufficient to trigger JH-mediated ovary development. In fact, the endocrine regulation of the CA during diapause is unlikely be as simple as the absence of a single factor [9]. It is possible that several additional factors and ETH signaling jointly regulate JH biosynthesis [9, 58]. We also have shown that injection of 20E results in an increase of *ETH* transcript levels in LD-induced females, and that dsEcR treatment decreases *ETH* mRNA level in SD-females. This is consistent with previous research, which showed that 20E acts as an important upstream regulator for *ETH* expression [24]. Therefore, photoperiod-regulated 20E signaling via *ETH* mediates JH production for reproductive diapause preparation. Compared to the known function of ETH in normal reproduction, our work clearly revealed a novel role of the 20E-ETH-JH axis in photoperiodic reproductive plasticity.

### 20E regulates lipid accumulation for diapause preparation in both JH-dependent and -independent manners

The hormone regulatory system could coordinate lipid accumulation and utilization during insect diapause [29, 59]. Previous studies from our laboratory showed that JH signaling suppresses diapause via inhibiting fat storage in the females of *C*. *bowringi* [10]. In this work, we found that 20E may serve as an upstream signaling of JH to reduce lipid storage in diapausing beetles. This finding would be a new mechanism of action where hormone signaling regulates energy metabolism during DPP. Additionally, our data suggested that 20E-inhibited lipid accumulation may also be independent of JH signaling as manipulating JH signaling could not fully eliminate the effects of 20E on lipid storage. One possibility is that the other endocrine factors may participate in the regulation of 20E on lipid metabolism during diapause of *C*. *bowringi*. A best candidate for such a factor is insulin/insulin-like peptide signaling [60]. More recent researches have focused on its regulatory mechanism in diapause and it is implicated as a primary regulator of insect diapause through influencing on energy storage and metabolic inhibition [61, 62]. The regulation of 20E on the insulin signaling has been well documented in the fat body of some insects, including *D*. *melanogaster* [63] and *B*. *mori* [64]. Hence, we speculate that this mechanism of which 20E regulates the insulin pathway might be involved in the diapause preparation process.

On the other hand, we also investigated which genes respond to 20E-JH signaling that play a role in regulation of lipid accumulation. Our data indicated that 20E could activate *Ptc* and *Smo* mediating hh signaling pathway in both JH-dependent and -independent manners to suppress lipid storage. But interestingly, 20E targets *Pangolin* and *β-catenin* mediating wnt signaling to regulate lipolysis process in JH-independent manner. A role of the hh and wnt signaling was established in energy homeostasis with a conserved role as a positive regulator of lipolysis [31, 32]. Although hh and wnt signaling are known to be under control of 20E during the developmental events [51, 65], we reveal here these relationships persist into the photoperiodic diapause. It is important to note that JH could also participate in regulation of 20E-hh signaling cascade, suggesting that regulation of lipid metabolism is extremely complex and diverse and occurs under control of different signaling pathways. Furthermore, we also determined that 20E-JH signaling could upregulate *Bmm* to promote lipolysis. The mechanisms of JH- and 20E-regulated *Bmm* in lipolysis have been reported in *Nilaparvata lugens* [66] and *B*. *mori* [64], respectively. This is a new discovery that 20E-JH signaling may be a vital regulatory pathway that functions to promote *Brummer* transcription in insects.

**Fig 8:**
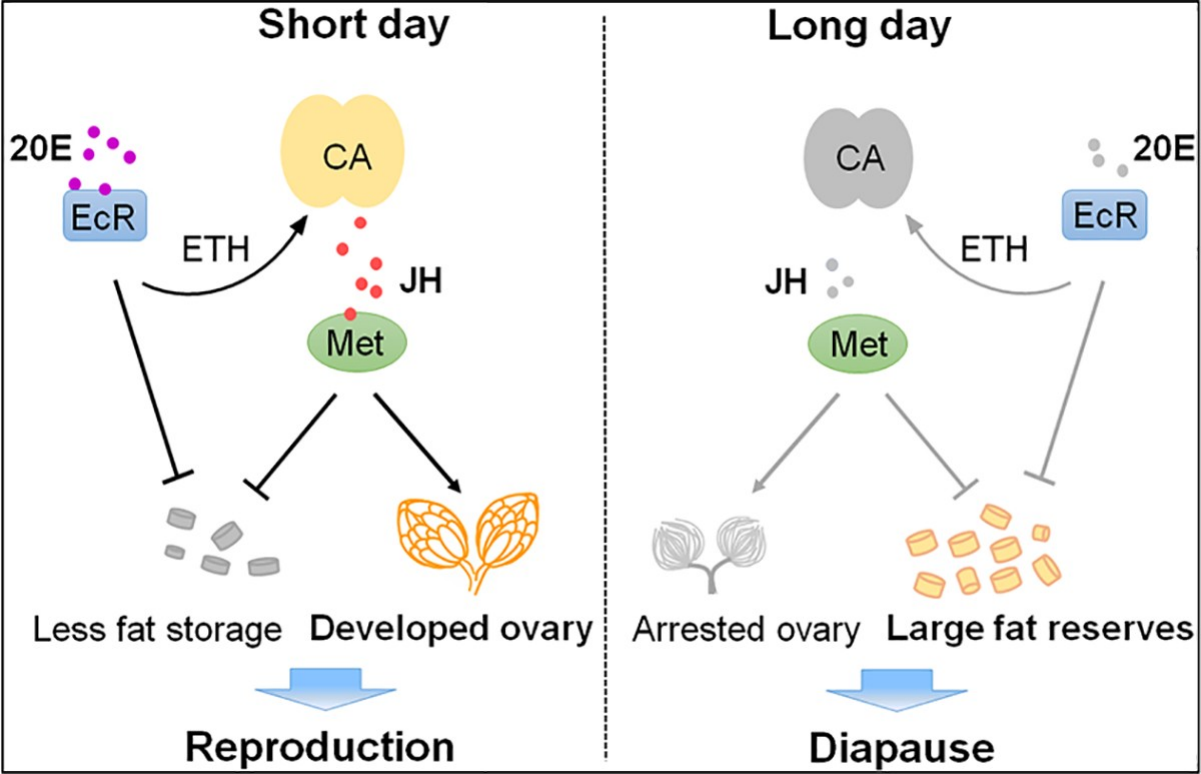
Model for 20E signaling in photoperiodic reproduction and diapause of *C*. *bowringi* females. 20E signaling under the short-day (SD; 12 h light, 12 h dark) condition, results in ovarian development via ETH-JH signaling. 20E-regulated JH signaling also inhibits lipid storage to block diapause preparation. Under the long-day (LD; 16 h light, 8 h dark) condition, 20E and ETH signaling is shut down and JH biosynthesis is inactivated, resulting in accumulation of lipid stores. In addition, 20E could suppress lipid accumulation and might be independent of JH signaling. Arrows represent promotion and T-bars represent inhibition. Gray and black represent the OFF and ON activity states of the genes or physiological processes, respectively.

In summary, this work strongly implicated that 20E signaling deficiency is essential to reproductive diapause preparation. We propose a model to depict the regulatory mechanism of the 20E-ETH-JH network regulating the occurrence of photoperiodic reproductive diapause in the cabbage beetle (Fig 8). Under the short-day photoperiod, 20E surge promotes vitellogenesis and ovarian development via ETH-JH signaling. Simultaneously, 20E signaling also inhibits lipid storage to block diapause in both JH-dependent and -independent manners. By contrast, in response to long-day photoperiod, the 20E and ETH signaling are shut down and JH biosynthesis is inhibited, leading to suppression of ovarian development and accumulation of huge fat reserves during diapause preparation. This endocrine network provides a new way of thinking to investigate the molecular mechanisms of regulation on insect reproductive plasticity.

## Materials and methods

### Ethic statement

The animal subjects used in the present study is a cabbage beetle, which is invertebrate and exempt from this requirement. No specific permits were required for the beetle’s collection from the field and for maintenance in laboratory. This study did not involve any endangered species, protected species or protected areas.

### Animals

Nearly 1000 *C*. *bowringi* beetles were originally collected from the natural population in Xiushui County (29°1′N, 114°4′E), Jiangxi Province, China in 2015 [67]. Offsprings of beetle that terminated diapause in 2017, 2018 and 2019 were used in this study. The beetles were reared at 25°C and 70% RH in an incubator (HP-250-GS; Wuhan Ruihua Instrument & Equipment, Hubei, China) and supplemented with radish leaves (*Raphanus sativus var*. *longipinnatus*) daily [26]. And they were kept at either of two photoperiod regimes: (*i*) Long-day (LD; 16 h light, 8 h dark) that allows reproductive diapause, and (*ii*) short-day (SD; 12 h light, 12 h dark) that induces adult reproduction [27]. The adults experienced LD and SD conditions during larval and pupal stages were diapause-destined (DD) and non-diapause-destined (NDD), respectively. The DPP of diapause-destined females, and the pre-oviposition phase (POP) of reproductive females, both occurred within the 4 days post eclosion (PE) period [26, 27]. After the DPP, DD adults will initiate diapause. All of the experiments in this study were performed by using the adult females which were during DPP or POP.

### RNA isolation and quantitative RT-PCR

Total RNA was isolated from different treatments using Trizol (Takara, D9108A, Japan) following the manufacturer’s instruction. First strand cDNA was reverse-transcribed using PrimeScript RT reagent Kit with gDNA Eraser (Takara, DRR047A, Japan) instructions. QRT-PCR was then performed with the corresponding primers (S5 Table) and SYBR Premix Ex Taq II (Takara, DRR081A, Japan) using an ABI QuantStudio 6 Flex (ThermoFisher Scientific, Massachusetts, USA). The *Rpl19* and *Actin1* were used as the reference genes for normalization of gene expression based on our previous work [67]. Relative expression was analyzed by the 2^*−ΔΔC*^_*T*_ method [68], based on three independent biological replicates and three technical replicates. The relative expression levels in the control groups were normalized to 1 and used to calibrate the data from the treatment groups. Relative expression levels represent fold changes between the treatment and control groups.

### 20E treatment and phenotype analysis

For the 20E induction, the original 20E (Selleck Chemicals, Houston, TX, USA) was dissolved in dimethyl sulfoxide (DMSO) to a concentration of 50 μg/μL. Then the solution was separately diluted to 2.5, 5, 10 and 25 μg/μL concentrations using 1× phosphate buffer saline (PBS) (HyClone, Logan, Utah, USA). Using a micro-injector (Nanolater2010, WPI), 200 nL of each 20E dilution was injected into diapause female beetles at 0 days PE (newly emerged without feeding) to reach the final 20E contents of 0.5, 1, 2 and 5 μg. Meanwhile, an equal volume of DMSO and 1× PBS was injected as the solvent control. We finally chose a concentration of 1 μg for the following experiments due to its favorable induction effect and low mortality (S9 Fig). To analyze the 20E-regulated phenotype, the images of the ovaries were collected using a stereo microscope fitted with a Nikon D5100 digital camera (Nikon, Tokyo, Japan), and the ovary size was determined by measuring the length and width with ScopePhoto 3.0 (Scopetek Opto Electric, Hangzhou, China) at 4 days PE. In addition, we graded the development of the ovaries to facilitate the statistical analysis of ovarian phenotypes (S10 Fig).

### ETH peptide treatment

The *ETH* (GenBank Accession No. MW291551) gene encodes two mature peptides, ETH1 (EEETPNFFLTAAKSVPRI-NH_2_) and ETH2 (SNKANSDFEKFFLKASKSVPRI-NH_2_) in *C*. *bowringi*. We used a mixture of ETH1 and ETH2 for all experiments; the concentration used for injections was based on previous studies [69, 70], albeit with some modifications. Briefly, the synthetic original ETH1 and ETH2 (GenScript corporation, Nanjing, China) were dissolved in 1×PBS to a concentration of 4000 μM, respectively. Then, the solution was separately diluted to 100, 200, 400, 1000 and 2000 μM concentrations. For injections, 200 nL ETH peptide was injected into newly emerged LD-female adults. Hence, 20, 40, 80, 200, and 400 pmol of ETH peptide was injected separately.

### RNAi experiments

RNAi experiments were performed according to our protocol previously described [10]. Briefly, dsRNA against *green fluorescent protein* (*GFP*), *methoprene-tolerant* (*Met*), *20E receptor* (*EcR*), *ecdysis-triggering hormone* (*ETH*) and *ecdysis-triggering hormone receptor* (*ETHR*) were synthesized using the corresponding primers (S6 Table) and a T7 transcription kit (ThermoFisher Scientific, Massachusetts, USA) according to the manufacturer’s instructions. Two μg of dsRNA in 200 nL was injected into the newly emerged female adults. An equal amount of dsRNA against *GFP* (dsGFP) was injected for use as a control. The ovarian phenotype was analyzed and tissues including ovaries (OV), fat bodies (FB), midguts (MG) and heads (HE) were collected at 4 days PE after dsRNA injection for gene expression analyses. For the dual-injection experiment, 1 μg of 20E was injected into the LD-induced females 24 h after dsRNA against *Met* (dsMet) injection. Analogously, 30 μg of JH analog methoprene (Sigma-Aldrich, St Louis, MO, USA) was treated with SD-induced females 24 h after the injection of dsRNA against *EcR* (dsEcR). The 400 pmol mature ETH peptide was injected into SD-induced females 24 h after dsETH injection.

### Triglyceride (TG) content and total lipid content measurement

Total TG content was determined using a Triglycerides Assay Kit (Nanjing Jiancheng Institute, Nanjing, China) according to the methods described previously [27]. There were four independent biological replicates for each treatment, each of which was analyzed with three technical replicates.

Total lipid content was estimated according to the previous studies [71] with some modifications. Briefly, the whole body was dried for 24 h at 100°C and measured on an electronic balance (OHAOS Company, New Jersey, USA), recorded as initial dry weight. Then lipids were extracted with 4 mL chloroform and methanol (2:1) solution for 24 h at room temperature. After further extraction with new chloroform and methanol solution for 6 h, dry weight was measured again. The difference between the initial dry weight and the dry weight of extracted lipids is considered to be the weight of the stored lipids. Total lipid content is defined as the percentage of weight of stored lipids to initial dry weight.

### Nile-Red staining

The Nile-red staining of fat bodies was performed by following the previous study [36]. Briefly, dissected fat bodies from 4-day female adults were fixed for 30 minutes in 4% paraformaldehyde (biosharp). Then tissues were washed twice with 1× PBS, incubated for 90 min in 1 μg/ml Nile-red (Sigma) and 25 min in 1 μg/ml DAPI (Sigma), and then washed twice with 1× PBS. All images were taken using a laser scanning microscopy (Leica TCS SP 8, Solms, Germany). The relative fluorescence intensity was analyzed with the ImageJ2x software (National Institute of Health, Bethesda, MD, USA).

### 20E determination

Hemolymph 30 μL was collected from LD- and SD-induced females at 4 days PE into tubes containing 150 μL methanol. The mixed solution was vortexed and centrifuged for 10 min at 12000g. The upper methanol layer was transferred to a new tube and dried completely by nitrogen. Then it was re-suspended in an EIA buffer, and subjected to enzyme immunoassay using the EIA kit (Cayman Chemical Co., Ann Arbor, MI, USA, A05120) to estimate the 20E titer according to the instructions.

### JH determination

The sample preparation was followed according to the previous descriptions with some modifications [72, 73]. In brief, each tube stored 50 μL of hemolymph with three replicates for different treatments. Methanol was added to make the ratio of methanol: hemolymph, 9: 1. The mixed solution was vortexed violently and permitted to stand at room temperature for 30 min, before centrifugation at 12000g for 15min. The upper methanol layer was transferred to a new tube and dried by nitrogen. It was then re-suspended in 50 μL methanol. JH III (Toronto Research Chemicals, Ontario, Canada) was dissolved in methanol.

Liquid chromatography-tandem mass spectrometry (LC-MS/MS) was used to quantify the JH titer of 20E treatment. The sample was separated on a reversed phase column (2.1×150 mm, 2.7 μm, poroshell 120 SB-C18) using gradient elution with methanol as a solvent A and 0.1% formic acid water as solvent B at a flow rate of 0.3 mL/min, utilizing an Agilent 1290 HPLC system with autosampler. The gradient was started at 20% A, decreased to 15% A at 4.5 min, followed by a decrease to 0% A at 7 min, increased to 20% A at 7.1 min, until held at 20% A at 10 min. Injection volume was 2 μL. MS/MS analysis was performed by electrospray ionization (ESI) in the positive mode on an SCIEX 6500Qtrap (Applied Biosystems, Massachusetts, USA). More detailed methods about the mass spectrometric parameters were referenced previously [73].

Similarly, the JH titer after gene knockdown was also quantified by LC-MS/MS tandem mass spectrometry. LC-MS/MS was carried out using a COSENSE-LCMS-8050 triple quadruple mass spectrometer (Shimadzu, Kyoto, Japan) equipped with an LC-30AD system (Shimadzu, Kyoto, Japan) and SIL-30AD autosampler (Shimadzu, Kyoto, Japan). A reversed phase column (Zorbax SB-Aq column, 100 mm×2.1 mm, 3.5 μm) (Agilent, Wilmington, DE, USA) using gradient elution with 0.1% formic acid water as a solvent A and methanol as solvent B at a flow rate of 0.3 mL/min. The gradient program is as follows: 0–1 min 40% B, 1–7 min 40% B to 100% B, 7–8 min 100% B, 8–8.1 min 100% B to 40% B and 8.1–10.5 min 40% B. Injection volume was 1 μL. More detailed methods about the mass spectrometric parameters were referenced previously [73].

### RNA-sequence analysis

We performed RNA-sequence (RNA-seq) analysis on the two issues including head and fat body on the 2^nd^ day after 20E injection by the Illumina HiSeq 2500 platform. Three biological replicates of each treatment were used for analysis. The DEGs were screened by DESeq using |log2 (fold change)| ≥ 1 and adjusted P-value< 0.05 for the screening parameters. The P-value was calculated as described by Robinson and Rivals [74, 75]. The FPKM (expected number of fragments per kilobase of transcript sequence per millions base pairs sequenced) method was used to calculate the expression counts of the unigenes and multiples of differential expression level between different samples for a gene [76]. We constructed the heat map analysis based on the FPKM values of genes which are focused on our research. Candidate unigenes were selected according to the NR annotation. RNA-seq data have been deposited in NCBI under accession number PRJNA575933.

### Statistics

All the data analysis was performed by using SPSS 11.5 (SPSS Inc., Chicago, IL, USA) and GraphPad Prism 8 (GraphPad Software Inc., San Diego, CA, USA) software packages. The mathematical significance of differences in expression levels of genes among different tissues was analyzed using one-way ANOVA followed by Tukey’s LSD tests (α = 0.05). In all other experiments, the significance of differences between samples was analyzed using the Independent-Samples t-test. *P < 0.05; **P < 0.01. Values are reported as means ± standard deviation (sd).

## Supporting information

S1 Fig Knocking down *EcR* inhibits ovarian development in SD-induced female *C*. *bowringi*. (A) The EcRA and EcRB protein sequences were deduced with the ExPASy Translate tool and the protein domains were predicted by the SMART tool. (B) Representative phenotypes of ovaries after dsGFP, dsEcR-2 and dsEcR-3 injection. (C) *EcR* knockdown efficiency in the fat bodies of the dsGFP control, *EcR* RNAi (dsEcR-2 and dsEcR-3). Relative gene expression levels in RNAi samples are shown as fold changes compared to the dsGFP control. (D) Tissue distribution of *EcR* in SD-treated females at 4 days PE. HE, head; MG, midgut; FB, fat body; OV, ovary. The relative gene expression levels in tissues are presented as fold changes as compared to the midguts. Different letters above bars indicate significant between-group differences determined by one-way ANOVA followed by Tukey’s LSD test (α = 0.05). Error bars represent the sd. **P < 0.01.
(TIF)

S2 Fig Effects of RNAi directed at 20E-biosynthetic genes on ovarian development and lipid storage. (A) The development grades and (B) size of ovaries were analyzed after treated with dsSpo, dsSad and dsShd. (C) RNAi efficiency of 20E biosynthetic genes and the changes in expression of *Vg1* and *Vg2* after RNAi treatment in SD-induced females, as determined by qRT-PCR. The expression levels of the Halloween and *Vg* genes were tested in the ovaries and fat bodies, respectively. Relative expression levels in the RNAi groups are presented as fold changes compared to the dsGFP control. (D) Relative levels of TG in the whole bodies on the 4^th^ day after the injections of dsSpo, dsSad and dsShd. Error bars represent the sd. *P < 0.05, **P < 0.01.
(TIF)

S3 Fig Relative expression of genes related to lipolysis and lipogenesis in the fat bodies of *C*. *bowringi* females, determined by qRT-PCR. Relative gene expression levels in the 20E treatment group are presented as fold changes compared to the control group (ethanol). Error bars represent the sd. *P < 0.05, **P < 0.01.
(TIF)

S4 Fig Expression patterns of *TGL1* in SD and LD females, and its RNAi efficiency. (A) The profiles of *TGL1* at 0, 2, and 4 days PE in the SD- and LD-induced females. Relative gene expression levels at various time points are presented as fold changes compared to the LD females at 0 days PE. (B) The tissue expression patterns of *TGL1* in SD-induced females at 4 days PE. Relative gene expression levels in tissue samples are presented as fold changes compared to the ovary samples. HE, head; MG, midgut; FB, fat body; OV, ovary. (C) RNAi efficiency of *TGL1* in the fat bodies of SD-induced females were tested at the 4 days after dsTGL1 injection. dsGFP injection served as a control. All the data were determined by qRT-PCR. Relative gene expression levels in *TGL1* RNAi are presented as fold changes compared to the dsGFP control. Individual letters above bars indicate significance between-group differences determined by one-way ANOVA followed by Tukey’s LSD test (α = 0.05). Error bars represent the sd. Asterisks indicate significant differences determined by an Independent-Samples t-test (*P < 0.05).
(TIF)

S5 Fig Relative expression of JH biosynthetic genes in the head and JH-inducible genes in the fat bodies of *C*. *bowringi* females, determined by qRT-PCR. Relative gene expression levels in the 20E group are represented as fold changes compared to the control (ethanol). Error bars represent the sd. *P < 0.05, **P < 0.01.
(TIF)

S6 Fig Disruption of *ETHR* expression leads to decreased yolk deposition and reduced ovary size in SD-induced females. (A) Relative mRNA levels of *ETHR* in four selected tissues from SD-females at 4 days PE. Relative gene expression levels in tissues are presented as fold changes compared to fat body samples. HE, head; MG, midgut; FB, fat body; OV, ovary. (B) Protein domains of *C*. *bowringi* ETHRA and ETHRB, predicted by the SMART tool. (C) Comparison of ovaries of dsETHR- and dsGFP-treated SD-females. (D) Knockdown efficiency after dsETHR injection. (E) The development grades and (F) ovary sizes were determined after treatment with dsETHR. (G) Transcriptional changes of *Vg1* and *Vg2* in the fat bodies after *ETHR* RNAi. (H) The changes in expression of genes related to JH biosynthesis and *Kr-h1* in the heads after dsETHR injection. Relative gene expression levels in *ETHR* RNAi samples are shown as fold changes compared to the dsGFP control (D, G, and H). Different letters above bars indicate significant between-group differences determined by one-way ANOVA followed by Tukey’s LSD test (α = 0.05). Error bars represent the sd. *P < 0.05, **P < 0.01.
(TIF)

S7 Fig Impaired reproductive phenotypes following reduction of *ETH* expression are rescued by mature ETH peptide in SD-induced females. (A) Representative examples of ovaries from SD-females after dsETH and 400 pmol ETH injection. (B) The development grades and (C) sizes of ovaries were analyzed after treated with dsETH and mature ETH peptide. (D) *Vg1* and *Vg2* transcript levels in the fat bodies of SD-females injected with dsETH and ETH peptide. (E) Reduced JH biosynthetic genes and *Kr-h1* expression levels in the heads following *ETH* knockdown and ETH peptide injection. The relative gene expression levels in the treatments are presented as fold changes compared to the dsGFP control. Error bars represent the sd. *P < 0.05, **P < 0.01.
(TIF)

S8 Fig Injection of ETH mature peptide in LD-induced females is insufficient to induce ovary development. (A) Yolk deposition, (B) development grades and sizes of ovaries were determined on the 4^th^ day after injections of a series of ETH peptide concentrations in LD-induced females.
(TIF)

S9 Fig 20E could induce the ovarian development of LD-induced females in a dose-dependent manner in *C*. *bowringi*. The 0.1, 0.5, 1, 2 and 5 μg of 20E were microinjected into the LD-induced females at 0 days PE to separately determine survival rate (Right Y axis) and the development grade of ovaries (Left Y axis) at 4 days PE.
(TIF)

S10 Fig The pattern diagrams of development grades of ovary in female *C*. *bowringi*. Grade 0: The ovary is small, white and nearly transparent, and differentiation of ovariole is not obvious; Grade I: Ovariole is clearly visible and inflated, and yolk deposition begins; Grade II: Rapid increase of oocyte size and yolk deposition; Grade III: Several mature eggs are visible in the ovariole, and the lateral oviduct are translucent; Grade IV: The mature eggs could be seen in the lateral oviduct.
(TIF)

S1 Table Expression of 20E biosynthesis genes at 0, 2 and 4 days PE in the LD- and SD-females of *C*. *bowringi*. (XLS)

S2 Table KEGG pathway analysis in DEGs. (XLS)

S3 Table Expression of genes related to lipolysis and lipogenesis after 20E injection in the fat bodies of *C*. *bowringi* LD-females. (XLS)

S4 Table Expression of JH biosynthesis and inducible genes after 20E injection in the LD-females of *C*. *bowringi*. (XLS)

S5 Table Primers for qRT-PCR. (PDF)

S6 Table Primers for dsRNA synthesis. (PDF)

## References

[1] Hand SC, Denlinger DL, Podrabsky JE, Roy R. Mechanisms of animal diapause: recent developments from nematodes, crustaceans, insects, and fish. Am J Physiol Regul Integr Comp Physiol. 2016;310(11):R1193–211. 10.1152/ajpregu.00250.2015 27053646 PMC4935499

[2] Denlinger DL. Regulation of diapause. Annu Rev Entomol. 2002;47:93–122. 10.1146/annurev.ento.47.091201.145137 11729070

[3] Hu C-K, Wang W, Brind’Amour J, Singh PP, Reeves GA, Lorincz MC, et al Vertebrate diapause preserves organisms long term through Polycomb complex members. Science. 2020;367(6480):870–4. 10.1126/science.aaw2601 32079766 PMC7532943

[4] Zhang XS, Wang T, Lin XW, Denlinger DL, Xu WH. Reactive oxygen species extend insect life span using components of the insulin-signaling pathway. Proc Natl Acad Sci U S A. 2017;114(37):E7832–E40. 10.1073/pnas.1711042114 28847950 PMC5604040

[5] Hahn DA, Denlinger DL. Energetics of insect diapause. Annu Rev Entomol. 2011;56:103–21. 10.1146/annurev-ento-112408-085436 20690828

[6] Hussein AM, Wang Y, Mathieu J, Margaretha L, Song C, Jones DC, et al Metabolic control over mTOR-dependent diapause-like state. Dev Cell. 2020;52(2):236–50. 10.1016/j.devcel.2019.12.018 31991105 PMC7204393

[7] Denlinger DL. Why study diapause? Entomol Res. 2008;38(1):1–9.

[8] Kostal V. Eco-physiological phases of insect diapause. J Insect Physiol. 2006;52(2):113–27. 10.1016/j.jinsphys.2005.09.008 16332347

[9] Denlinger DL, Yocum GD, Rinehart JP. Hormonal control of diapause In: Gilbert LI (Elsevier, Amsterdam), editor. Insect Endocrinology; 2012 pp. 430–463.

[10] Liu W, Li Y, Zhu L, Zhu F, Lei C-L, Wang X-P. Juvenile hormone facilitates the antagonism between adult reproduction and diapause through the methoprene-tolerant gene in the female *Colaphellus bowringi*. Insect Biochem Mol Biol 2016;74:50–60. 10.1016/j.ibmb.2016.05.004 27180724

[11] Bajgar A, Jindra M, Dolezel D. Autonomous regulation of the insect gut by circadian genes acting downstream of juvenile hormone signaling. Proc Natl Acad Sci U S A. 2013;110(11):4416–21. 10.1073/pnas.1217060110 23442387 PMC3600444

[12] Urbanova V, Bazalova O, Vaneckova H, Dolezel D. Photoperiod regulates growth of male accessory glands through juvenile hormone signaling in the linden bug, *Pyrrhocoris apterus*. Insect Biochem Mol Biol. 2016;70:184–90. 10.1016/j.ibmb.2016.01.003 26826599

[13] Tawfik AI, Tanaka Y, Tanaka S. Possible involvement of ecdysteroids in photoperiodically induced suppresion of ovarian development in a Japanese strain of the migratory locust, *Locusta migratoria*. J Insect Physiol 2002;48(4):411–8. 10.1016/s0022-1910(02)00058-6 12770090

[14] Zachardová D, Sehnal F, Landa V. Makisterone A content and gondadal development in *Pyrrhocoris apterus* reared under long versus short photoperiods In: Tonner M, Soldán T, Bennettová B, editors. Regulation of Insect Reproduction IV. Czechoslovak Academy of Sciences Praha; 1989pp. 59–71.

[15] Kozlova T, Thummel CS. Steroid regulation of postembryonic development and reproduction in *Drosophila*. Trends Endocrinol Metab. 2000;11(7):276–80. 10.1016/s1043-2760(00)00282-4 10920384

[16] Lafont R, Dauphin-Villemant C, Warren JT, Rees H. Ecdysteroid chemistry and biochemistry In: Gilbert LI, latrou K, Gill SS (Elsevier, Amsterdam), editors. Insect Endocrinology.; 2005 pp. 125–195.

[17] Lagueux M, Hirn M, Hoffmann JA. Ecdysone during ovarian development in *Locusta migratoria*. J Insect Physiol. 1977;23(1):109–19. 10.1016/0022-1910(77)90116-0 858928

[18] Martin D, Wang SF, Raikhel AS. The vitellogenin gene of the mosquito *Aedes aegypti* is a direct target of ecdysteroid receptor. Mol Cell Endocrinol. 2001;173(1–2):75–86. 10.1016/s0303-7207(00)00413-5 11223179

[19] Swevers L, Iatrou K. The ecdysone regulatory cascade and ovarian development in lepidopteran insects: insights from the silkmoth paradigm. Insect Biochem Mol Biol. 2003;33(12):1285–97. 10.1016/j.ibmb.2003.06.012 14599500

[20] Parthasarathy R, Sheng Z, Sun Z, Palli SR. Ecdysteroid regulation of ovarian growth and oocyte maturation in the red flour beetle, *Tribolium castaneum*. Insect Biochem Mol Biol. 2010;40(6):429–39. 10.1016/j.ibmb.2010.04.002 20385235 PMC2916939

[21] Swevers L. An update on ecdysone signaling during insect oogenesis. Curr Opin Insect Sci. 2019;31:8–13. 10.1016/j.cois.2018.07.003 31109678

[22] Dubrovsky EB. Hormonal cross talk in insect development. Trends Endocrinol Metab. 2005;16(1):6–11. 10.1016/j.tem.2004.11.003 15620543

[23] Gu SH, Chow YS. Regulation of juvenile hormone biosynthesis by ecdysteroid levels during the early stages of the last two larval instars of *Bombyx mori*. J Insect Physiol 1996;42(7):625–32.

[24] Areiza M, Nouzova M, Rivera-Perez C, Noriega FG. 20-Hydroxyecdysone stimulation of juvenile hormone biosynthesis by the mosquito corpora allata. Insect Biochem Mol Biol. 2015;64:100–5. 10.1016/j.ibmb.2015.08.001 26255691 PMC4558257

[25] Meiselman M, Lee SS, Tran RT, Dai H, Ding Y, Rivera-Perez C, et al Endocrine network essential for reproductive success in *Drosophila melanogaster*. Proc Natl Acad Sci U S A. 2017; 114(19):E3849–E3858. 10.1073/pnas.1620760114 28439025 PMC5441734

[26] Xue F, Spieth HR, Li Aq, Ai H. The role of photoperiod and temperature in determination of summer and winter diapause in the cabbage beetle, *Colaphellus bowringi* (Coleoptera: Chrysomelidae). J Insect Physiol. 2002;48(3):279–86. 10.1016/s0022-1910(01)00172-x 12770101

[27] Tan QQ, Feng L, Liu W, Zhu L, Lei CL, Wang XP. Differences in the pre-diapause and pre-oviposition accumulation of critical nutrients in adult females of the beetle *Colaphellus bowringi*. Entomol Exp Appl. 2016;160(2):117–25.

[28] Niwa R, Niwa YS. Enzymes for ecdysteroid biosynthesis: their biological functions in insects and beyond. Biosci Biotech Bioch. 2014;78(8):1283–92. 10.1080/09168451.2014.942250 25130728

[29] Liu W, Tan QQ, Zhu L, Li Y, Zhu F, Lei CL, et al Absence of juvenile hormone signalling regulates the dynamic expression profiles of nutritional metabolism genes during diapause preparation in the cabbage beetle *Colaphellus bowringi*. Insect Mol Biol. 2017;26(5):530–42. 10.1111/imb.12316 28544235

[30] Wang X, Hou Y, Saha TT, Pei G, Raikhel AS, Zou Z. Hormone and receptor interplay in the regulation of mosquito lipid metabolism. Proc Natl Acad Sci U S A. 2017;114(13):E2709–E2718 10.1073/pnas.1619326114 28292900 PMC5380040

[31] Pospisilik JA, Schramek D, Schnidar H, Cronin SJ, Nehme NT, Zhang X, et al *Drosophila* genome-wide obesity screen reveals hedgehog as a determinant of brown versus white adipose cell fate. Cell. 2010;140(1):148–60. 10.1016/j.cell.2009.12.027 20074523

[32] Longo KA, Wright WS, Kang S, Gerin I, Chiang S-H, Lucas PC, et al Wnt10b inhibits development of white and brown adipose tissues. J Biol Chem. 2004;279(34):35503–9. 10.1074/jbc.M402937200 15190075

[33] Tan QQ, Liu W, Zhu F, Lei CL, Wang XP. Fatty acid synthase 2 contributes to diapause preparation in a beetle by regulating lipid accumulation and stress tolerance genes expression. Sci Rep. 2017;7:40509 10.1038/srep40509 28071706 PMC5223116

[34] Ziouzenkova O, Orasanu G, Sharlach M, Akiyama TE, Berger JP, Viereck J, et al Retinaldehyde represses adipogenesis and diet-induced obesity. Nat Med. 2007;13(6):695–702. 10.1038/nm1587 17529981 PMC2233696

[35] Arrese EL, Soulages JL. Insect fat body: energy, metabolism, and regulation. Annu Rev Entomol. 2010;55:207–25. 10.1146/annurev-ento-112408-085356 19725772 PMC3075550

[36] Zhu L, Tian Z, Guo S, Liu W, Zhu F, Wang XP. Circadian clock genes link photoperiodic signals to lipid accumulation during diapause preparation in the diapause-destined female cabbage beetles *Colaphellus bowringi*. Insect Biochem Mol Biol. 2019;104:1–10. 10.1016/j.ibmb.2018.11.001 30423421

[37] Wang S, Liu S, Liu H, Wang J, Zhou S, Jiang RJ, et al 20-hydroxyecdysone reduces insect food consumption resulting in fat body lipolysis during molting and pupation. J Mol Cell Biol. 2010;2(3):128–38. 10.1093/jmcb/mjq006 20430856

[38] Smykal V, Bajgar A, Provaznik J, Fexova S, Buricova M, Takaki K, et al Juvenile hormone signaling during reproduction and development of the linden bug, *Pyrrhocoris apterus*. Insect Biochem Mol Biol. 2014;45:69–76. 10.1016/j.ibmb.2013.12.003 24361539

[39] Kayukawa T, Minakuchi C, Namiki T, Togawa T, Yoshiyama M, Kamimura M, et al Transcriptional regulation of juvenile hormone-mediated induction of *Kruppel homolog 1*, a repressor of insect metamorphosis. Proc Natl Acad Sci U S A. 2012;109(29):11729–34. 10.1073/pnas.1204951109 22753472 PMC3406821

[40] Zhu L, Yin TY, Sun D, Liu W, Zhu F, Lei CL, et al Juvenile hormone regulates the differential expression of putative juvenile hormone esterases via methoprene-tolerant in non-diapause-destined and diapause-destined adult female beetle. Gene. 2017;627:373–8. 10.1016/j.gene.2017.06.061 28679117

[41] Rivera-Perez C, Nouzova M, Lamboglia I, Noriega FG. Metabolic analysis reveals changes in the mevalonate and juvenile hormone synthesis pathways linked to the mosquito reproductive physiology. Insect Biochem Mol Biol. 2014;51:1–9. 10.1016/j.ibmb.2014.05.001 24833260 PMC4107215

[42] Noriega FG. Juvenile hormone biosynthesis in insects: What is new, what do we know, and what questions remain? Int Sch Res Notices. 2014;2014:967361 10.1155/2014/967361 27382622 PMC4897325

[43] Zitnan D, Zitnanova I, Spalovska I, Takac P, Park Y, Adams ME. Conservation of ecdysis-triggering hormone signalling in insects. J Exp Biol. 2003;206(Pt 8):1275–89. 10.1242/jeb.00261 12624163

[44] Di Cara F, King-Jones K. The circadian clock is a key driver of steroid hormone production in *Drosophila*. Curr Biol. 2016;26(18):2469–77. 10.1016/j.cub.2016.07.004 27546572

[45] Saunders DS. Dormancy, diapause, and the role of the circadian system in insect photoperiodism. Annu Rev Entomol. 2020;65:373–89. 10.1146/annurev-ento-011019-025116 31594413

[46] Poupardin R, Schottner K, Korbelova J, Provaznik J, Dolezel D, Pavlinic D, et al Early transcriptional events linked to induction of diapause revealed by RNAseq in larvae of drosophilid fly, *Chymomyza costata*. BMC genomics. 2015;16:720 10.1186/s12864-015-1907-4 26391666 PMC4578651

[47] Ameku T, Niwa R. Mating-induced increase in germline stem cells via the neuroendocrine system in female *Drosophila*. PLoS Genet. 2016;12(6):e1006123 10.1371/journal.pgen.1006123 27310920 PMC4911108

[48] Richard DS, Watkins NL, Serafin RB, Gilbert LI. Ecdysteroids regulate yolk protein uptake by *Drosophila melanogaster* oocytes. J Insect Physiol. 1998;44(7):637–44. 10.1016/s0022-1910(98)00020-1 12769946

[49] Wu Q, Jiang Z, Bai C. The role of ecdysteroids in the reproductive diapause of *Dermacentor niveus* Neumann. Insect Sci. 1994;1(2):164–71.

[50] Kamoshida Y, Fujiyama-Nakamura S, Kimura S, Suzuki E, Lim J, Shiozaki-Sato Y, et al Ecdysone receptor (EcR) suppresses lipid accumulation in the *Drosophila* fat body via transcription control. Biochem Biophys Res Commun. 2012;421(2):203–7. 10.1016/j.bbrc.2012.03.135 22503687

[51] Chen W, Xu WH. Wnt/beta-catenin signaling regulates *Helicoverpa armigera* pupal development by up-regulating c-Myc and AP-4. Insect Biochem Mol Biol. 2014;53:44–53. 10.1016/j.ibmb.2014.07.004 25038464

[52] Roy S, Saha TT, Johnson L, Zhao B, Ha J, White KP, et al Regulation of gene expression patterns in mosquito reproduction. PLoS Genet. 2015;11(8):e1005450 10.1371/journal.pgen.1005450 26274815 PMC4537244

[53] Parthasarathy R, Sun Z, Bai H, Palli SR. Juvenile hormone regulation of vitellogenin synthesis in the red flour beetle, *Tribolium castaneum*. Insect Biochem Mol Biol. 2010;40(5):405–14. 10.1016/j.ibmb.2010.03.006 20381616 PMC2875371

[54] Whisenton LR, Bowen MF, Granger NA, Gilbert LI, Bollenbacher WE. Brain-mediated 20-hydroxyecdysone regulation of juvenile hormone synthesis by the corpora allata of the tobacco hornworm, *Manduca sexta*. Gen Comp Endocrinol. 1985;58(2):311–8. 10.1016/0016-6480(85)90347-8 3996893

[55] Liu S, Li K, Gao Y, Liu X, Chen W, Ge W, et al Antagonistic actions of juvenile hormone and 20-hydroxyecdysone within the ring gland determine developmental transitions in *Drosophila*. Proc Natl Acad Sci U S A. 2018;115(1):139–44. 10.1073/pnas.1716897115 29255055 PMC5776822

[56] Hult EF, Huang J, Marchal E, Lam J, Tobe SS. RXR/USP and EcR are critical for the regulation of reproduction and the control of JH biosynthesis in *Diploptera punctata*. J Insect Physiol. 2015;80:48–60. 10.1016/j.jinsphys.2015.04.006 25917982

[57] Roma GC, Bueno OC, Camargo-Mathias MI. Morpho-physiological analysis of the insect fat body: a review. Micron. 2010;41(5):395–401. 10.1016/j.micron.2009.12.007 20206534

[58] Kang DS, Denlinger DL, Sim C. Suppression of allatotropin simulates reproductive diapause in the mosquito *Culex pipiens*. J Insect Physiol. 2014;64:48–53. 10.1016/j.jinsphys.2014.03.005 24657669 PMC4150688

[59] Sim C, Denlinger DL. Juvenile hormone III suppresses forkhead of transcription factor in the fat body and reduces fat accumulation in the diapausing mosquito, *Culex pipiens*. Insect Mol Biol. 2013;22(1):1–11. 10.1111/j.1365-2583.2012.01166.x 23121109

[60] Matsunaga Y, Honda Y, Honda S, Iwasaki T, Qadota H, Benian GM, et al Diapause is associated with a change in the polarity of secretion of insulin-like peptides. Nat Commun. 2016;7:10573 10.1038/ncomms10573 26838180 PMC4742890

[61] Williams KD, Busto M, Suster ML, So AK, Ben-Shahar Y, Leevers SJ, et al Natural variation in *Drosophila melanogaste*r diapause due to the insulin-regulated PI3-kinase. Proc Natl Acad Sci U S A. 2006;103(43):15911–5. 10.1073/pnas.0604592103 17043223 PMC1635102

[62] Sim C, Denlinger DL. Insulin signaling and FOXO regulate the overwintering diapause of the mosquito *Culex pipiens*. Proc Natl Acad Sci U S A. 2008;105(18):6777–81. 10.1073/pnas.0802067105 18448677 PMC2373331

[63] Rusten TE, Lindmo K, Juhasz G, Sass M, Seglen PO, Brech A, et al Programmed autophagy in the *Drosophila* fat body is induced by ecdysone through regulation of the PI3K pathway. Dev Cell. 2004;7(2):179–92. 10.1016/j.devcel.2004.07.005 15296715

[64] Hossain MS, Liu Y, Zhou S, Li K, Tian L, Li S. 20-Hydroxyecdysone-induced transcriptional activity of FoxO upregulates *brummer* and *acid lipase-1* and promotes lipolysis in *Bombyx* fat body. Insect Biochem Mol Biol. 2013;43(9):829–38. 10.1016/j.ibmb.2013.06.007 23811219

[65] Li T-R, White KP. Tissue-specific gene expression and ecdysone-regulated genomic networks in *Drosophila*. Dev Cell. 2003;5(1):59–72. 10.1016/s1534-5807(03)00192-8 12852852

[66] Lu K, Zhou J, Chen X, Li W, Li Y, Cheng Y, et al Deficiency of brummer impaires lipid mobilization and JH-mediated vitellogenesis in the brown planthopper, *Nilaparvata lugens*. Front Physiol. 2018;9:1535 10.3389/fphys.2018.01535 30425657 PMC6218678

[67] Tan QQ, Zhu L, Li Y, Liu W, Ma WH, Lei CL, et al A de novo transcriptome and valid reference genes for quantitative real-time PCR in *Colaphellus bowringi*. PLoS ONE. 2015;10(2):e0118693 10.1371/journal.pone.0118693 25692689 PMC4334893

[68] Schmittgen TD, Livak KJ. Analyzing real-time PCR data by the comparative C(T) method. Nat Protoc 2008;3(6):1101–8. 10.1038/nprot.2008.73 18546601

[69] Meiselman MR, Kingan TG, Adams ME. Stress-induced reproductive arrest in *Drosophila* occurs through ETH deficiency-mediated suppression of oogenesis and ovulation. BMC Biol. 2018;16(1):18 10.1186/s12915-018-0484-9 29382341 PMC5791332

[70] Lenaerts C, Cools D, Verdonck R, Verbakel L, Vanden Broeck J, Marchal E. The ecdysis triggering hormone system is essential for successful moulting of a major hemimetabolous pest insect, Schistocerca gregaria. Sci Rep. 2017;7:46502 10.1038/srep46502 28417966 PMC5394484

[71] Morita A, Soga K, Hoson T, Kamisaka S, Numata H. Changes in mechanical properties of the cuticle and lipid accumulation in relation to adult diapause in the bean bug, *Riptortus clavatus*. J Insect Physiol. 1999;45(3):241–7. 10.1016/s0022-1910(98)00119-x 12770371

[72] Cornette R, Gotoh H, Koshikawa S, Miura T. Juvenile hormone titers and caste differentiation in the damp-wood termite *Hodotermopsis sjostedti* (Isoptera, Termopsidae). J Insect Physiol. 2008;54(6):922–30. 10.1016/j.jinsphys.2008.04.017 18541259

[73] Zhao B, Hou Y, Wang J, Kokoza VA, Saha TT, Wang XL, et al Determination of juvenile hormone titers by means of LC-MS/MS/MS and a juvenile hormone-responsive Gal4/UAS system in *Aedes aegypti* mosquitoes. Insect Biochem Mol Biol. 2016;77:69–77. 10.1016/j.ibmb.2016.08.003 27530057 PMC5028310

[74] Robinson MD, Grigull J, Mohammad N, Hughes TR. FunSpec: a web-based cluster interpreter for yeast. BMC Bioinformatics. 2002;3:35 10.1186/1471-2105-3-35 12431279 PMC139976

[75] Rivals I, Personnaz L, Taing L, Potier MC. Enrichment or depletion of a GO category within a class of genes: which test? Bioinformatics. 2007;23(4):401–7. 10.1093/bioinformatics/btl633 17182697

[76] Trapnell C, Williams BA, Pertea G, Mortazavi A, Kwan G, Van Baren MJ, et al Transcript assembly and quantification by RNA-Seq reveals unannotated transcripts and isoform switching during cell differentiation. Nat Biotechnol. 2010;28(5):511–5. 10.1038/nbt.1621 20436464 PMC3146043

