# Supplementary figures and images for: Steroid hormone ecdysone deficiency stimulates preparation for photoperiodic reproductive diapause
Source: PLoS Genet. 2021 Feb 2;17(2):e1009352. doi: 10.1371/journal.pgen.1009352 (PMC7880476; doi:10.1371/journal.pgen.1009352)

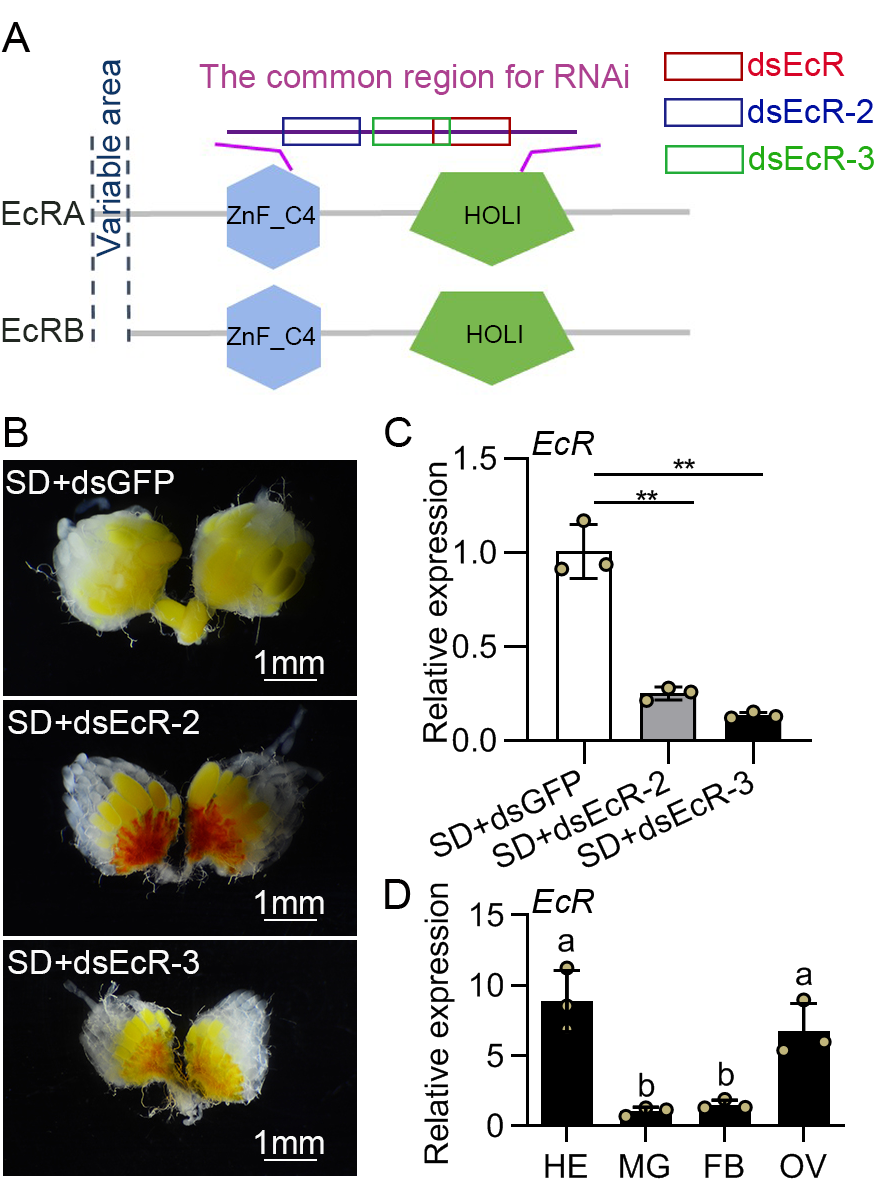

Supplement: S1 Fig — (A) The EcRA and EcRB protein sequences were deduced with the ExPASy Translate tool and the protein domains were predicted by the SMART tool. (B) Representative phenotypes of ovaries after dsGFP, dsEcR-2 and dsEcR-3 injection. (C) EcR knockdown efficiency in the fat bodies of the dsGFP control, EcR RNAi (dsEcR-2 and dsEcR-3). Relative gene expression levels in RNAi samples are shown as fold changes compared to the dsGFP control. (D) Tissue distribution of EcR in SD-treated females at 4 days PE. HE, head; MG, midgut; FB, fat body; OV, ovary. The relative gene expression levels in tissues are presented as fold changes as compared to the midguts. Different letters above bars indicate significant between-group differences determined by one-way ANOVA followed by Tukey’s LSD test (α = 0.05). Error bars represent the sd. **P < 0.01. (TIF) [file pgen.1009352.s001.tif]

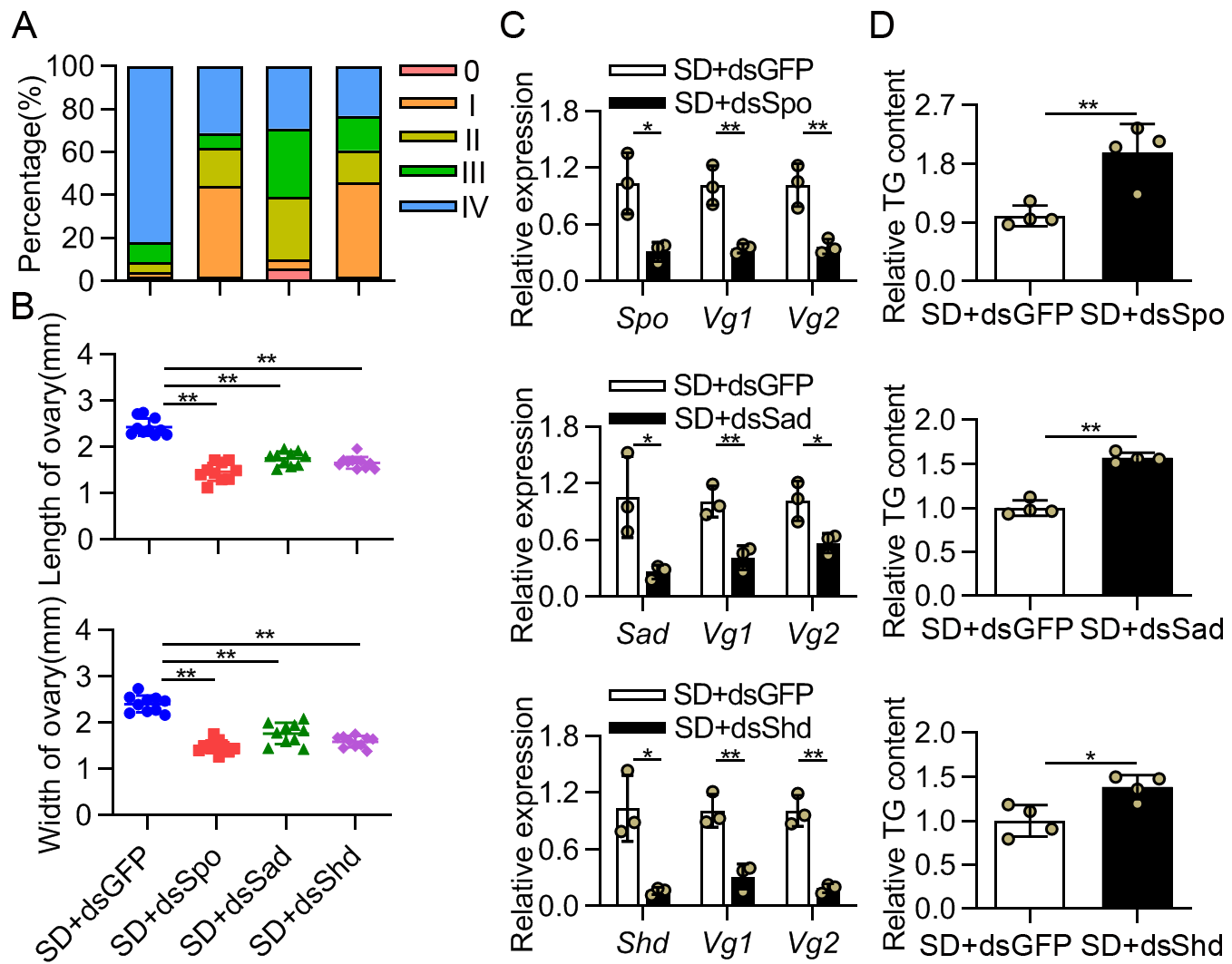

Supplement: S2 Fig — (A) The development grades and (B) size of ovaries were analyzed after treated with dsSpo, dsSad and dsShd. (C) RNAi efficiency of 20E biosynthetic genes and the changes in expression of Vg1 and Vg2 after RNAi treatment in SD-induced females, as determined by qRT-PCR. The expression levels of the Halloween and Vg genes were tested in the ovaries and fat bodies, respectively. Relative expression levels in the RNAi groups are presented as fold changes compared to the dsGFP control. (D) Relative levels of TG in the whole bodies on the 4th day after the injections of dsSpo, dsSad and dsShd. Error bars represent the sd. *P < 0.05, **P < 0.01. (TIF) [file pgen.1009352.s002.tif]

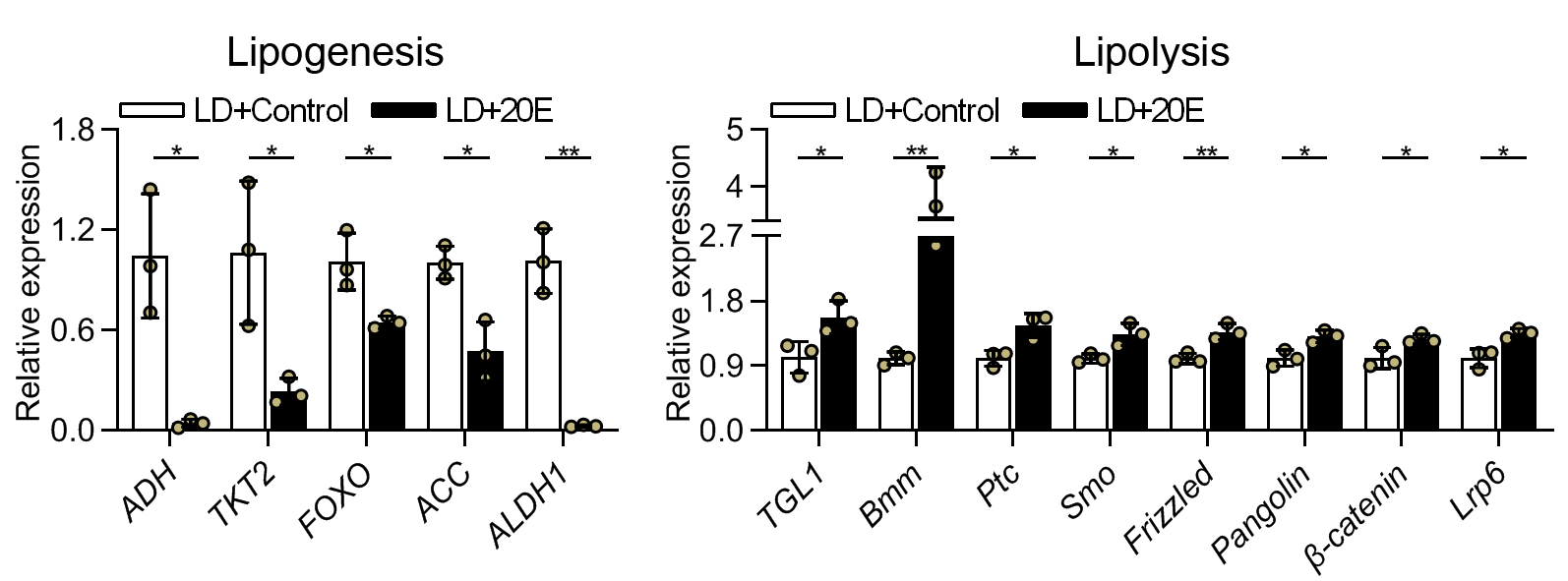

Supplement: S3 Fig — Relative gene expression levels in the 20E treatment group are presented as fold changes compared to the control group (ethanol). Error bars represent the sd. *P < 0.05, **P < 0.01. (TIF) [file pgen.1009352.s003.tif]

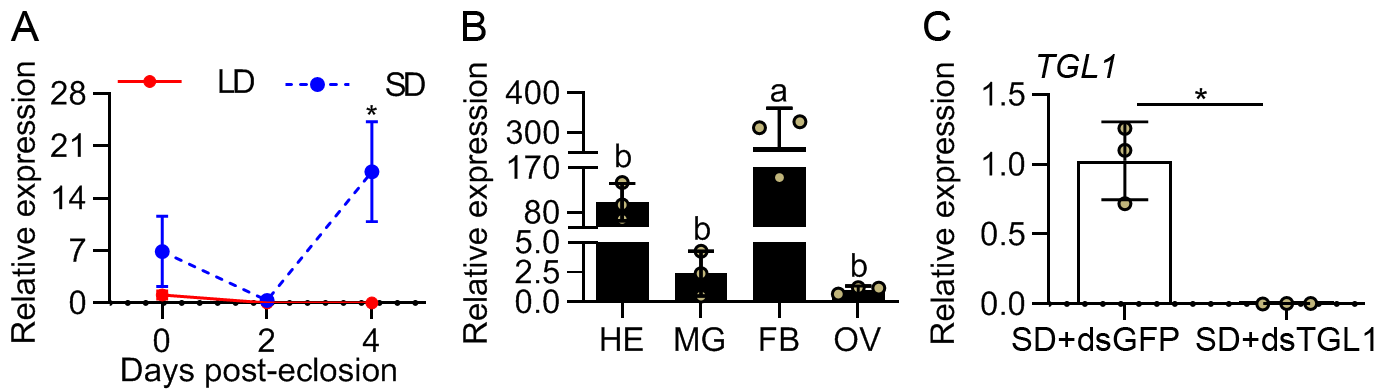

Supplement: S4 Fig — (A) The profiles of TGL1 at 0, 2, and 4 days PE in the SD- and LD-induced females. Relative gene expression levels at various time points are presented as fold changes compared to the LD females at 0 days PE. (B) The tissue expression patterns of TGL1 in SD-induced females at 4 days PE. Relative gene expression levels in tissue samples are presented as fold changes compared to the ovary samples. HE, head; MG, midgut; FB, fat body; OV, ovary. (C) RNAi efficiency of TGL1 in the fat bodies of SD-induced females were tested at the 4 days after dsTGL1 injection. dsGFP injection served as a control. All the data were determined by qRT-PCR. Relative gene expression levels in TGL1 RNAi are presented as fold changes compared to the dsGFP control. Individual letters above bars indicate significance between-group differences determined by one-way ANOVA followed by Tukey’s LSD test (α = 0.05). Error bars represent the sd. Asterisks indicate significant differences determined by an Independent-Samples t-test (*P < 0.05). (TIF) [file pgen.1009352.s004.tif]

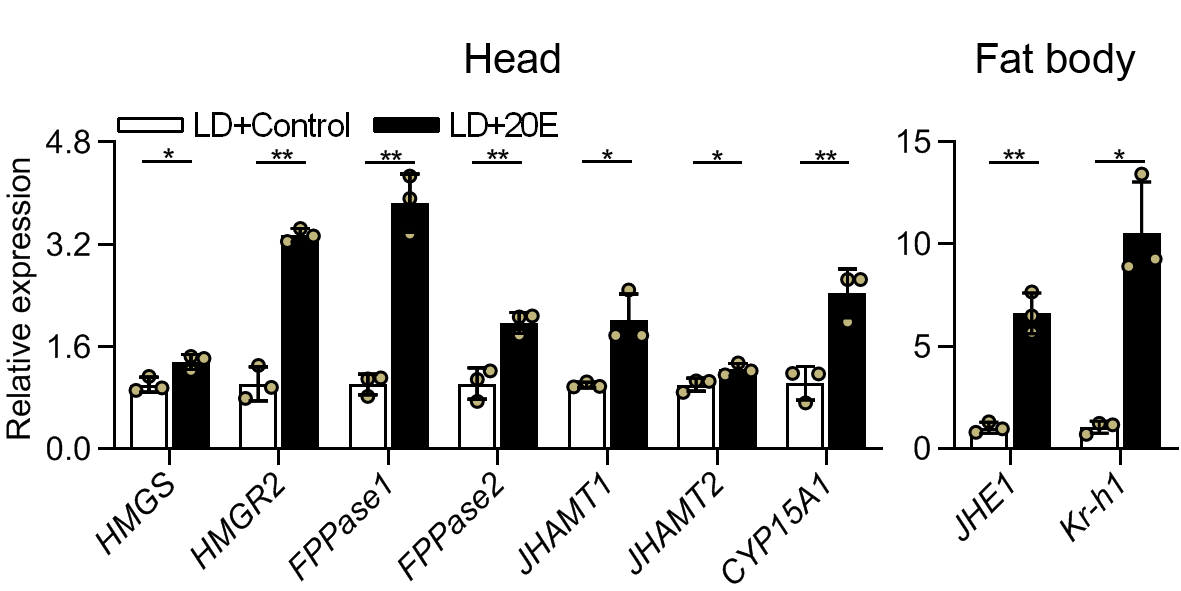

Supplement: S5 Fig — Relative gene expression levels in the 20E group are represented as fold changes compared to the control (ethanol). Error bars represent the sd. *P < 0.05, **P < 0.01. (TIF) [file pgen.1009352.s005.tif]

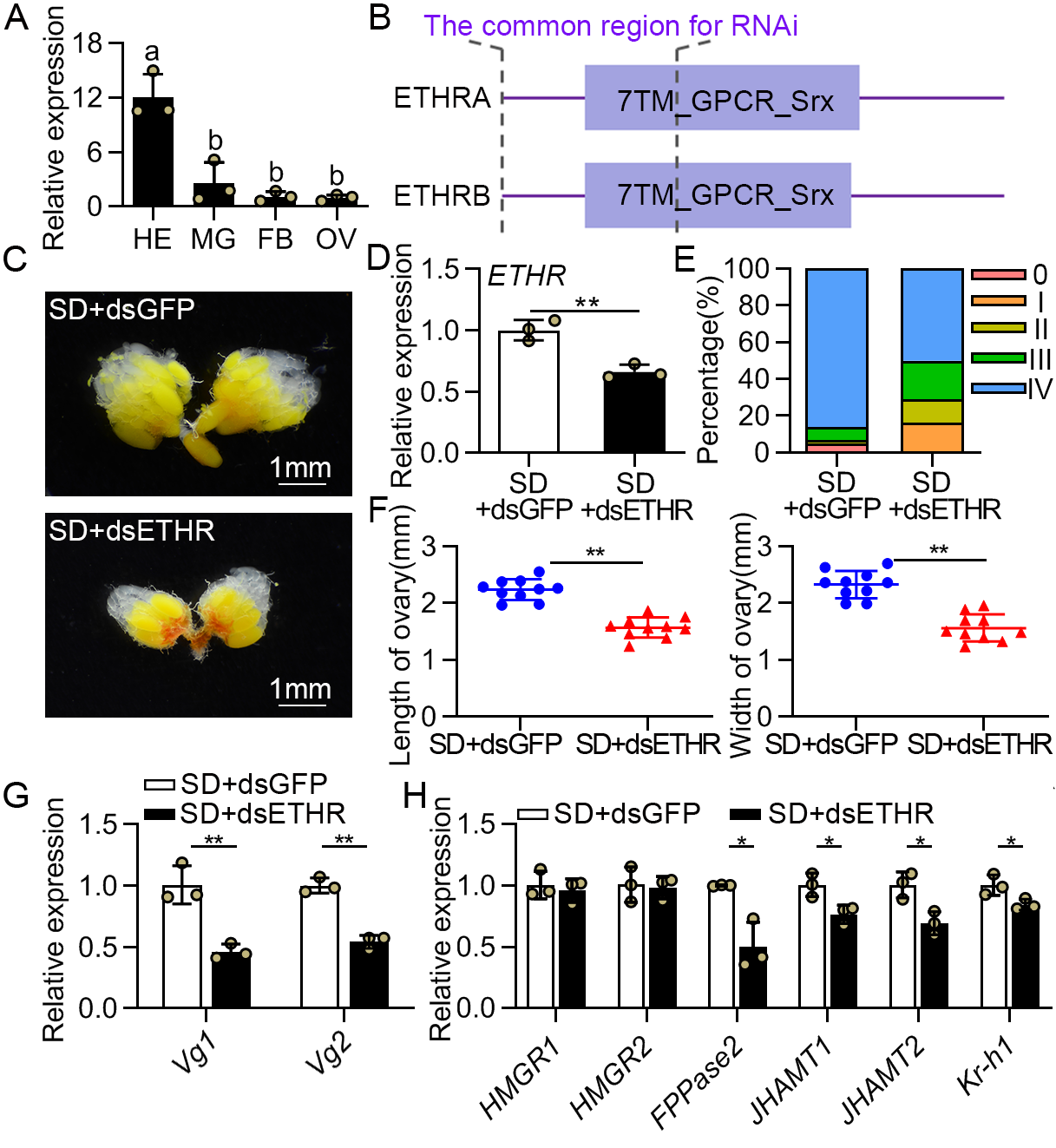

Supplement: S6 Fig — (A) Relative mRNA levels of ETHR in four selected tissues from SD-females at 4 days PE. Relative gene expression levels in tissues are presented as fold changes compared to fat body samples. HE, head; MG, midgut; FB, fat body; OV, ovary. (B) Protein domains of C. bowringi ETHRA and ETHRB, predicted by the SMART tool. (C) Comparison of ovaries of dsETHR- and dsGFP-treated SD-females. (D) Knockdown efficiency after dsETHR injection. (E) The development grades and (F) ovary sizes were determined after treatment with dsETHR. (G) Transcriptional changes of Vg1 and Vg2 in the fat bodies after ETHR RNAi. (H) The changes in expression of genes related to JH biosynthesis and Kr-h1 in the heads after dsETHR injection. Relative gene expression levels in ETHR RNAi samples are shown as fold changes compared to the dsGFP control (D, G, and H). Different letters above bars indicate significant between-group differences determined by one-way ANOVA followed by Tukey’s LSD test (α = 0.05). Error bars represent the sd. *P < 0.05, **P < 0.01. (TIF) [file pgen.1009352.s006.tif]

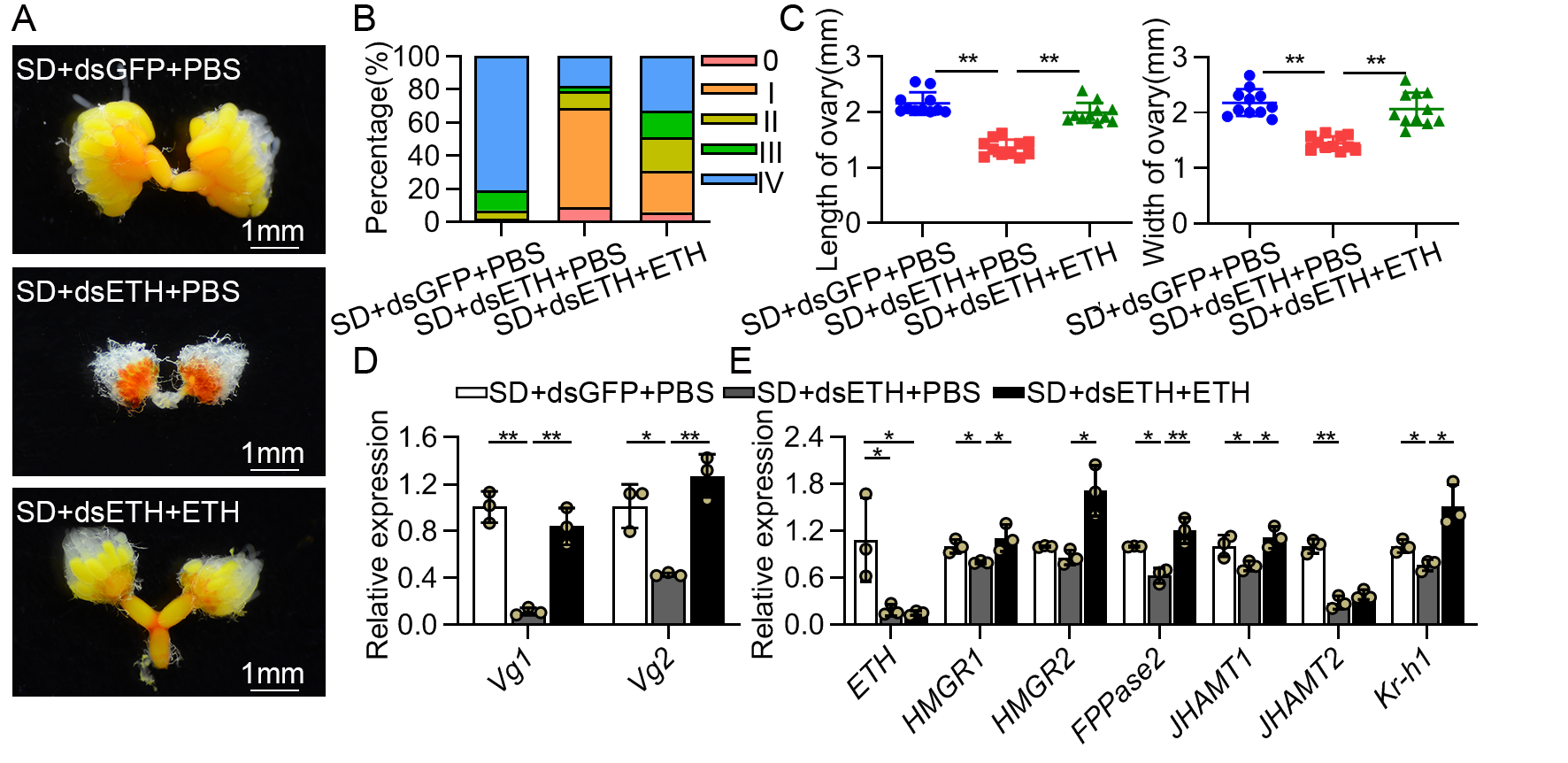

Supplement: S7 Fig — (A) Representative examples of ovaries from SD-females after dsETH and 400 pmol ETH injection. (B) The development grades and (C) sizes of ovaries were analyzed after treated with dsETH and mature ETH peptide. (D) Vg1 and Vg2 transcript levels in the fat bodies of SD-females injected with dsETH and ETH peptide. (E) Reduced JH biosynthetic genes and Kr-h1 expression levels in the heads following ETH knockdown and ETH peptide injection. The relative gene expression levels in the treatments are presented as fold changes compared to the dsGFP control. Error bars represent the sd. *P < 0.05, **P < 0.01. (TIF) [file pgen.1009352.s007.tif]

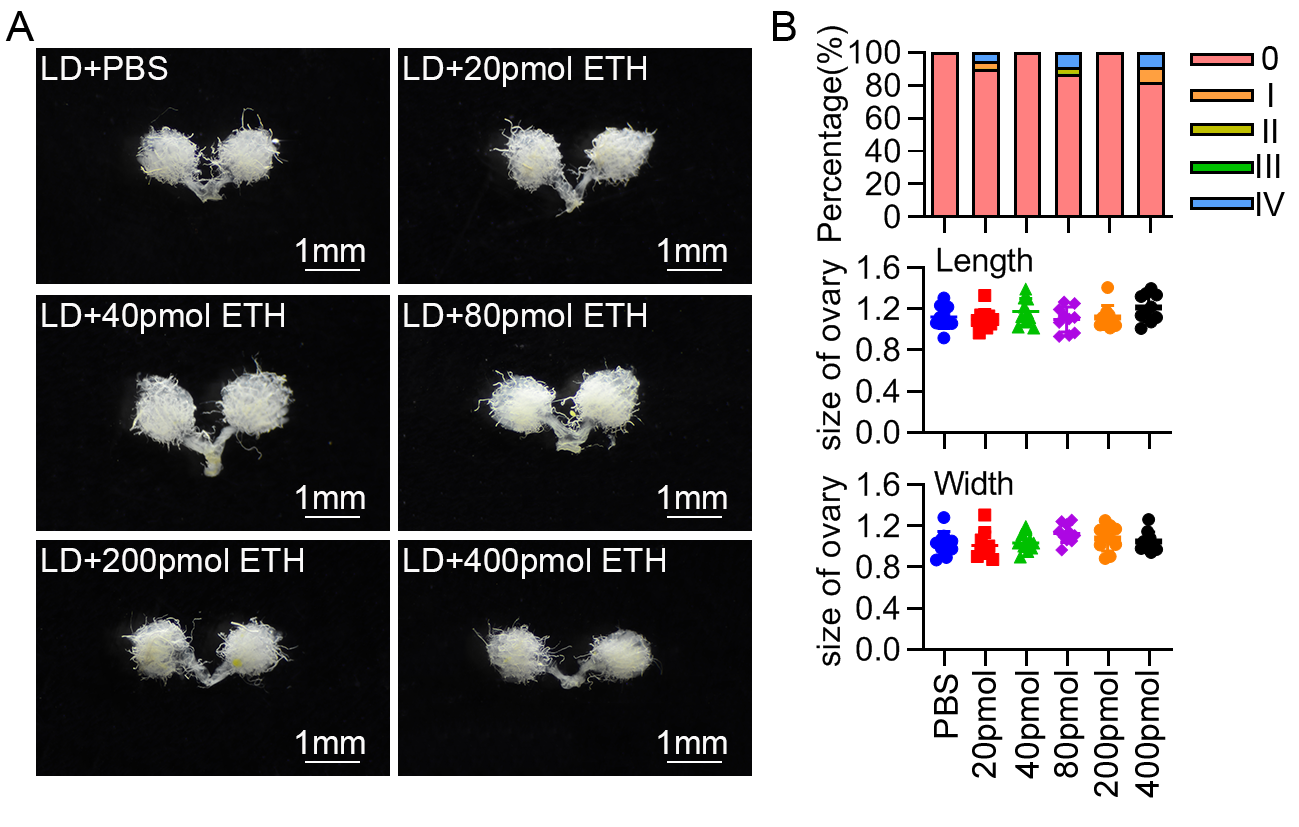

Supplement: S8 Fig — (A) Yolk deposition, (B) development grades and sizes of ovaries were determined on the 4th day after injections of a series of ETH peptide concentrations in LD-induced females. (TIF) [file pgen.1009352.s008.tif]

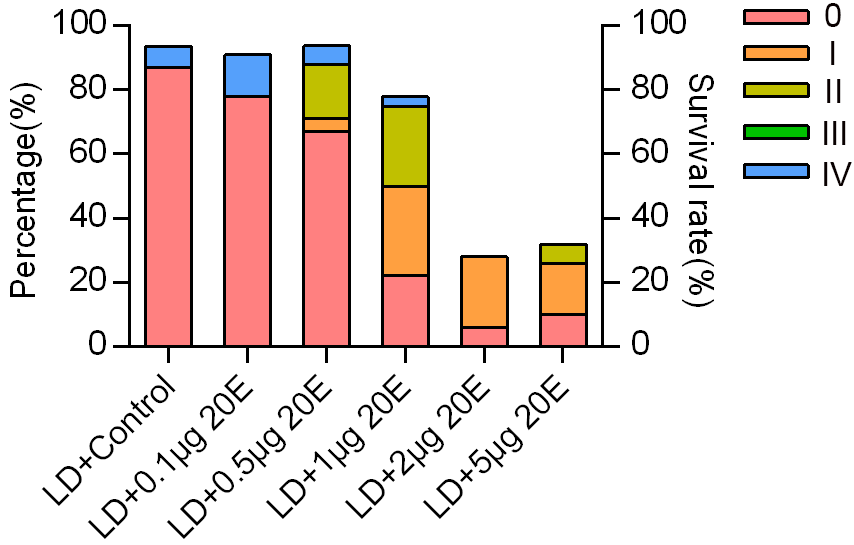

Supplement: S9 Fig — The 0.1, 0.5, 1, 2 and 5 μg of 20E were microinjected into the LD-induced females at 0 days PE to separately determine survival rate (Right Y axis) and the development grade of ovaries (Left Y axis) at 4 days PE. (TIF) [file pgen.1009352.s009.tif]

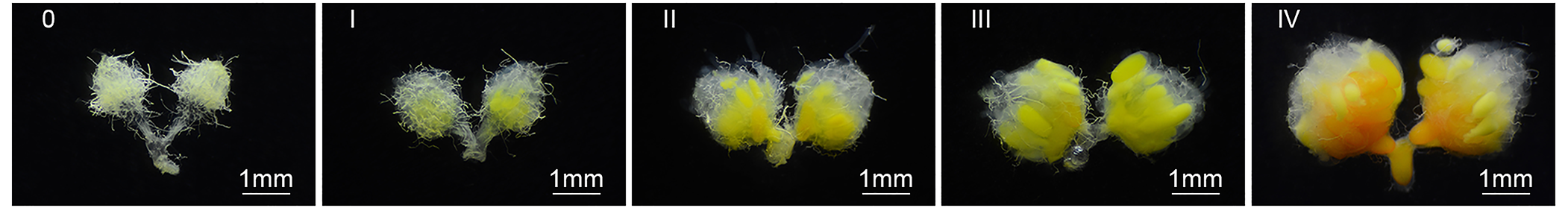

Supplement: S10 Fig — Grade 0: The ovary is small, white and nearly transparent, and differentiation of ovariole is not obvious; Grade I: Ovariole is clearly visible and inflated, and yolk deposition begins; Grade II: Rapid increase of oocyte size and yolk deposition; Grade III: Several mature eggs are visible in the ovariole, and the lateral oviduct are translucent; Grade IV: The mature eggs could be seen in the lateral oviduct. (TIF) [file pgen.1009352.s010.tif]
