# Supplementary material for: Steroid hormone ecdysone deficiency stimulates preparation for photoperiodic reproductive diapause
Source: PLoS Genet. 2021 Feb 2;17(2):e1009352. doi: 10.1371/journal.pgen.1009352 (PMC7880476; doi:10.1371/journal.pgen.1009352)
Supplement: S5 Table — (PDF) [file pgen.1009352.s015.pdf]

Table S5. Primers for qRT-PCR.

Genes	Forward primers (5'–3')	Reverse primers (5'–3')	PCR efficiency (%)	Standard curve R^2
Rpl19	gtaatgcgatgcggcaagaa	aaacctgtagcggtgcactc	102.2	0.9980
Actin1	tcaagcggtgttagctctgg	tatcgatcacgatgccggtg	100.2	0.9960
EcR	cggcatgggtgaaacaatcg	ttccagccttcgatcagagc	92.0	0.9910
Spook	tccgaaaccatggccactat	tgacaaccacacagggtgaa	98.2	0.9981
Phantom	tcggacgatacaggattccca	ttgctagctcgtcaccaaca	97.1	0.9940
Shadow	gcctgcctttgatcggtact	aggatccgcaacaaaaacgc	99.9	0.9987
Shade	actgacgagcccaaaaacca	acgacgtttcgagacccaat	106.0	0.9996
Kr-h1	caactcaaagtgcacacccg	ccgtaatgcgcaacctgatg	102.0	0.9980
JHE1	ggtagtgccagcatgacgaa	gctttgcaatccgtttgtcg	109.0	0.9780
JHE2	cctgccagtgacattttggc	tcatttggcgcaactttcgg	99.9	0.9970
Vg1	ggaatgcgcgtgagattgac	cgaagcagttttgtggggtg	100.2	0.9960
Vg2	tggaccgattcacaaccctg	tcgaagatgaggaatgcggg	105.4	0.9970
ADH	ctgacctcgtgcagttttgc	gatgttgacgacgatggggt	91.2	0.9970
TGL1	gaatactggcaaggtggcct	acgtcgtatccttcgtctgc	96.0	0.9970
ALDH1	ggcaaaagtccttgcgttgt	cgaatgttctcgaagcagcg	99.7	0.9940
TKT2	ggtgggcaaggccaatatct	actctaacgccagttcctgc	96.1	0.9940
HMGR1	tggttttcacacttcactcaaact	tggtacggaagattttggct	108.2	0.9956
HMGR2	tagcaacactgacgctgaca	acagtagaggacagccagaca	106.4	0.9964
HMGS	aataaccaataacgggaagg	actgaacgactgactctc	98.1	0.9981
FPPase1	tactgcggacattggaggtt	attcgggttggtactctgtgg	104.4	0.9956
FPPase2	ggacccttaatcgccaacca	actgccctttgggatcagt	93.9	0.9898
JHAMT1	gggttttcagtgtagattctgga	cgaaaatcagtttgtccctctgt	91.51	0.9980
JHAMT2	ccgtcgagagtcgaaatgga	acaagtttcagcacttacatcataa	101.7	0.9840
CYP15A1	actgggaatcgcacataggg	tccctcctccaaaaggaacg	95.8	0.9953
FOXO	ccttcctacccccactctga	gcccatcaactgacccatca	95.2	0.9910
ACC	tgagatcggtacgtcgatgg	ttccgcattggctttcaggt	93.1	1.0000
Brummer	aaccgcacaagagcgtgata	ggccgaatctctgtccaaca	90.6	0.9990
Patched	actcatcggccaaaaccaca	ctttggttgtgtaagggccac	87.4	0.9980
Smo	aacaacactacctgcatgggg	tgtcttcgcattttggcatgt	86.6	0.9980
Ci	cctcgcatctcaagcatcca	ccagtcgtagcagtggacaa	90.9	0.9980
Frizzled	tgtcgctcttcaggatacgc	caatcagagcgggcaatgtg	90.0	0.9900
TCF	atgaagaagtgcagagcccg	cgtctgattggttgccatcg	94.0	1.0000
β-catenin	gttccatctctgacgggcaa	caggaaacatggcagcccta	89.0	1.0000
Lrp6	cgttatcgcccttaccgtca	gccggttcgccatcatatct	101.0	0.9900
ETH	aattgtgaggaggaaaccccaa	tacttcgacctattcggggca	108.7	0.9880
ETHR	gcgagaagaggacatctacca	tgtgagggacacgactgttg	102.0	0.9900
