# Supplementary material for: Steroid hormone ecdysone deficiency stimulates preparation for photoperiodic reproductive diapause
Source: PLoS Genet. 2021 Feb 2;17(2):e1009352. doi: 10.1371/journal.pgen.1009352 (PMC7880476; doi:10.1371/journal.pgen.1009352)
Supplement: S6 Table — (PDF) [file pgen.1009352.s016.pdf]

Table S6. Primers for dsRNA synthesis.

**Genes**	**Forward primers (5'–3')**	**Reverse primers (5'–3')**
Spo	gcgtaatacgactcactataggccgctcctagagatttcacg	gcgtaatacgactcactataggggttcgtcccaataaccctt
Sad	gcgtaatacgactcactataggaccagcgagcttcaactcat	gcgtaatacgactcactataggcaccacattcctcaacaacg
Shd	gcgtaatacgactcactataggattttggactgccagtttgg	gcgtaatacgactcactataggataccgttcggtgctggtag
EcR	gcgtaatacgactcactataggaacgaatacgaacaccctgc	gcgtaatacgactcactataggagcagagcatactcggcatt
Met	gcgtaatacgactcactataggatgattgaggaagtgtcggg	gcgtaatacgactcactatagggattctcgtggtggaccagt
GFP	gcgtaatacgactcactataggtggtcccaattctcgtggaac	gcgtaatacgactcactataggcttgaagttgaccttgatgcc
EcR-2	gcgtaatacgactcactatagggtcgagaagacctctcccct	gcgtaatacgactcactatagggtacttccggtaccacgcat
EcR-3	gcgtaatacgactcactataggacagcaccacgaatggatct	gcgtaatacgactcactataggagcttgtcgaagcctggtaa
TGL1	gcgtaatacgactcactatagggaccagtcccttgttgagga	gcgtaatacgactcactataggggtaccatgctgccgtactt
ETH	gcgtaatacgactcactatagggtgaggaggaaaccccaaat	gcgtaatacgactcactataggtaaaagttgacaggaccggc
ETHR	gcgtaatacgactcactataggatagaaccgaacgcaaccac	gcgtaatacgactcactatagggaggcagataagcgatgctc
